# Spatial analysis of stromal signatures identifies invasive front carcinoma-associated fibroblasts as suppressors of anti-tumor immune response in esophageal cancer

**DOI:** 10.1186/s13046-023-02697-y

**Published:** 2023-05-31

**Authors:** Jian-Zhong He, Yang Chen, Fa-Min Zeng, Qing-Feng Huang, Hai-Feng Zhang, Shao-Hong Wang, Shuai-Xia Yu, Xiao-Xiao Pang, Ye Liu, Xiu-E Xu, Jian-Yi Wu, Wen-Jun Shen, Zhan-Yu Li, En-Min Li, Li-Yan Xu

**Affiliations:** 1 Guangdong Provincial Key Laboratory of Infectious Diseases and Molecular Immunopathology, Institute of Oncologic Pathology, Shantou University Medical College, Shantou, 515041 Guangdong People’s Republic of China; 2 Department of Pathology, The Fifth Affiliated Hospital, Sun Yat-Sen University, Zhuhai, 519000 Guangdong Province People’s Republic of China; 3 Department of Pathology, First People’s Hospital of Yunnan Province, Kunming, 650032 Yunnan Province China; 4 Cancer Research Center, Shantou University Medical College, Shantou, 515041 Guangdong People’s Republic of China; 5 Department of Pathology and Laboratory Medicine, University of British Columbia, Vancouver, British Columbia V5Z 1L3 Canada; 6 Departments of Pathology, Shantou Central Hospital, Shantou, 515041 Guangdong People’s Republic of China; 7 Department of Pathology, Sichuan Academy of Medical Sciences and Sichuan Provincial People’s Hospital, Chinese Academy of Sciences Sichuan Translational Medicine Research Hospital, Chengdu, 610072 China; 8 Key Laboratory of Molecular Biology for High Cancer Incidence Coastal Chaoshan Area, Department of Biochemistry and Molecular Biology, Shantou University Medical College, Shantou, 515041 Guangdong People’s Republic of China; 9 Department of Bioinformatics, Shantou University Medical College, Shantou, 515041 Guangdong People’s Republic of China

**Keywords:** TME, Carcinoma-associated fibroblast, Macrophage, Prognostic model, Esophageal squamous cell carcinoma

## Abstract

**Background:**

Increasing evidence indicates that the tumor microenvironment (TME) is a crucial determinant of cancer progression. However, the clinical and pathobiological significance of stromal signatures in the TME, as a complex dynamic entity, is still unclear in esophageal squamous cell carcinoma (ESCC).

**Methods:**

Herein, we used single-cell transcriptome sequencing data, imaging mass cytometry (IMC) and multiplex immunofluorescence staining to characterize the stromal signatures in ESCC and evaluate their prognostic values in this aggressive disease. An automated quantitative pathology imaging system determined the locations of the lamina propria, stroma, and invasive front. Subsequently, IMC spatial analyses further uncovered spatial interaction and distribution. Additionally, bioinformatics analysis was performed to explore the TME remodeling mechanism in ESCC. To define a new molecular prognostic model, we calculated the risk score of each patient based on their TME signatures and pTNM stages.

**Results:**

We demonstrate that the presence of fibroblasts at the tumor invasive front was associated with the invasive depth and poor prognosis. Furthermore, the amount of α-smooth muscle actin (α-SMA)^+^ fibroblasts at the tumor invasive front positively correlated with the number of macrophages (MØs), but negatively correlated with that of tumor-infiltrating granzyme B^+^ immune cells, and CD4^+^ and CD8^+^ T cells. Spatial analyses uncovered a significant spatial interaction between α-SMA^+^ fibroblasts and CD163^+^ MØs in the TME, which resulted in spatially exclusive interactions to anti-tumor immune cells. We further validated the laminin and collagen signaling network contributions to TME remodeling. Moreover, compared with pTNM staging, a molecular prognostic model, based on expression of α-SMA^+^ fibroblasts at the invasive front, and CD163^+^ MØs, showed higher accuracy in predicting survival or recurrence in ESCC patients. Regression analysis confirmed this model is an independent predictor for survival, which also identifies a high-risk group of ESCC patients that can benefit from adjuvant therapy.

**Conclusions:**

Our newly defined biomarker signature may serve as a complement for current clinical risk stratification approaches and provide potential therapeutic targets for reversing the fibroblast-mediated immunosuppressive microenvironment.

**Supplementary Information:**

The online version contains supplementary material available at 10.1186/s13046-023-02697-y.

## Background

Accumulating evidence has demonstrated that the tumor microenvironment (TME) is a crucial determinant of tumor growth, invasion, and metastasis, and also influences cancer patient response to treatments [1–4]. The TME is a complex and dynamic entity that contains various types of cells, such as fibroblasts, macrophages (MØs), mast cells, lymphocytes and endotheliocytes, and continually changes over the course of disease progression [2]. Despite our increasing understanding of how tumor cells interact with the TME to facilitate tumor initiation and dissemination, thereby influencing patient prognosis [2, 5], the relationship between different stromal cells within the TME, and their pathobiological significance, remain uncharacterized in esophageal squamous cell carcinoma (ESCC) [6, 7]. ESCC, one of the most common and deadliest carcinomas, is the most common histologic subtype of esophageal cancer in China [6, 7]. The dismal 5-year survival rate for ESCC patients is due to recurrence, metastasis and lack of response to adjuvant therapy [8]. Therefore, to identify the key prognostic markers and effective therapeutic targets, it is important to investigate the stromal components and understand their significance in ESCC.

Fibroblasts, one of the main stromal components in malignant solid tumors, play a crucial role in promoting tumor progression and serve as potential therapeutic targets [1]. Carcinoma-associated fibroblasts (CAFs), as activated fibroblasts, have been shown to upregulate the expression of α-SMA to increase the secretion of growth factors and elicit multiple functions in cancer [1, 2]. However, the clinical significance of CAFs in cancer remains contradictory, as CAFs have been shown to associate with either favorable or unfavorable outcome in different cancers [9]. Recent studies suggest that CAFs contain different subsets with distinct origins and functions [1, 9, 10], and secrete diverse cytokines and extracellular matrix (ECM) to carry out a multitude of roles [1, 9]. In breast cancer, the presence of CAFs itself does not determine the aggressiveness of tumors, but rather a CD10^+^GPR77^+^ subset of CAFs driven by NF-κB signaling plays a key role in promoting tumor formation and chemoresistance [11]. More importantly, CAFs, as the dominating component of the TME, not only impact the progression of cancer, but also participate in TME remodeling and inflammatory cell activation [1, 9].

Although CAFs in ESCC have been shown to secrete VEGF to induce the formation of a pre-metastatic niche by recruiting bone marrow cells [5], the precise role of CAFs in ESCC remains largely unexplored. Herein, we sought to comprehensively characterize the tissue distribution and significance of stromal components in ESCC patient samples. We identified a subpopulation of CAFs at the invasive front that are associated with ESCC progression and poor prognosis. We found that CAFs at the invasive front of ESCC tumors are correlated with the density of MØ infiltration, and conspire to impede immune cell effector function and antitumor immune responses. In addition, we define a novel prognostic model, with the characteristics of stromal components, that shows superior predictive power than the current TNM staging system, and is capable of estimating the effectiveness of adjuvant therapy in ESCC patients. This prognostic model may serve as an effective complement for the current clinical risk stratification system, and be used to determine whether adjuvant therapy would be beneficial.

## Methods

### Single-cell transcriptome sequencing data processing

The raw single-cell RNA-seq data ((RJNA777911) in this study was obtained from 11 ESCC patients who underwent curative resection at the Cancer Hospital of Shantou University Medical College in 2018 [12]. None of the patients had been treated with any antitumor therapy prior to tumor resection, and the main clinical information is summarized in Supplementary Table S1. All expression data were normalized prior to analysis, and the detailed information has been reported in our previous article [12]. Using Cell Ranger software, the sequencing data were aligned to the hg38 reference genome. The number of cells, UMI (unique molecular identifier) counts, and genes detected per cell were obtained. Based on the distribution of UMIs, a cellular gene expression matrix was generated and analyzed with Seurat R package (version 4.2.0). Cells with > 200 and < 6000 genes detected were retained for downstream analyses. The feature counts for each cell were divided by the total counts for that cell and multiplied by the scale factor of 10^4^, and then natural log transformed. For dimensionality reduction, the most variable genes were determined using the FindVariableFeatures function with default parameters. Dimensionality reduction was then performed using PCA and UMAP with the first 17 PCs as input, determined by visualizing the drop off in PC variance explained by using the ElbowPlot function.

### Cell type annotation

In order to determine cell types, we performed unsupervised clustering using the FindClusters function in Seurat. We annotated cell clusters with the dominant expression pattern of canonical cell markers. Specifically, we used the following cell markers for annotation of cell clusters: Epithelial/Tumor: EPCAM, keratin genes (KRT7, KRT8, KRT17), SPRR3; T cells: CD3D/E/G; CD4^+^ T cells: CD4; CD8^+^ T cells: CD8A/B; myeloid cells: LYZ, CD86, CD68, FCGR3A; monocytes: CD14; macrophages: CD68, CD163, C1QA; dendritic cells: HLA-DR, CD1A/B/C, CD40; B cells and plasma cells: CD19, CD79A/B, MS4A1, CD38; NK cells: NCAM1, FCGR3A/B; endothelial cells: CLDN5, FLT1, PECAM1, RAMP2; mast cells: TPSAB1, MS4A2; and stromal cells: DCN, C1R, COL1A1, ACTA2, VIM. We first utilized the FindClusters function at resolution 0.8 to identify the broad cell types based on the above markers. Subset annotations of T, myeloid and stromal cells were determined by isolating their respective clusters, repeating unsupervised clustering at resolution 0.8 and annotation with canonical cell markers of each group of cells. For stromal cell types, clusters highly expressing α-SMA (e.g. median expression > 0) were inferred to be α-SMA^+^, and all others were inferred to be α-SMA¯. 

### Cell–cell communication

To systematically investigate the cell–cell communications between α-SMA^+^ CAFs, α-SMA^−^ CAFs and other cell types, we analyzed the expression levels of ligand–receptor interacting pairs within cell types using CellChat R package (version 1.1.3). In brief, we first loaded the normalized expression matrix into CellChat and applied the preprocessing functions ‘identifyOverExpressedGenes’, ‘identifyOverExpressedInteractions’, and ‘projectData’ with default parameters. Next, we applied the core functions ‘computeCommunProb’, ‘filterCommunication’, ‘computeCommunProbPathway’ and ‘aggregateNet’ with default parameters to compute the communication strength between any interacting cell types and identify all the signaling pathways with significant communications.

### Differential expression analysis and functional enrichment analysis

We used FindMarkers in Seurat package to find differentially expressed genes between subsets of α-SMA^+^ CAFs and α-SMA^−^ CAFs. It was run with cutoff logFC 0.25 of a subset compared to the rest. We used ggplot2 R package to visualize the differentially-expressed genes through the log-fold change expression and the difference in the percentage of cells expressing the genes comparing α-SMA^+^ CAFs versus α-SMA^−^ CAFs (Δ Percentage Difference).

For the functional enrichment study, genes highly expressing (fold change > 1.5) and statistically significant differences (*P* < 0.05) between α-SMA^+^ CAFs and α-SMA^−^ CAFs were identified and examined for relevant functional annotations using the Metascape tool (https://metascape.org). The functional annotations used were: KEGG Pathway, GO Biological Processes, Reactome Gene Sets, Canonical Pathways, CORUM, WikiPathways, and the PANTHER Pathway.

### Imaging mass cytometry

IMC staining was performed according to a standard protocol (PN00322 A3). Briefly, 5 μm-thin FFPE tissue sections were collected, dewaxed and rehydrated. Subsequently, antigen retrieval was conducted in antigen retrieval buffer (Agilent, Cat#S236784-2, PH9) in a water-bath at 96℃ for 30 min. After cooling and rinsing with ddH_2_O, the sections were blocked with 3% BSA in DPBS for 45 min at room temperature. Then, the sections were incubated overnight at 4℃ with metal-conjugated antibody cocktails diluted in DPBS/0.5% BSA (Supplementary Table S2). After washing twice with DPBS/0.1% Triton X-100 (Thermo Scientific, Cat#85,111) and DPBS, the slides were exposed to DNA intercalator (Fluidigm, Cat#201192A) for 30 min at room temperature. Finally, the slides were rinsed twice in ddH_2_O and air-dried before IMC analysis.

For IMC data acquisition, the tissue sections were scanned by a pulsed deep UV laser shot and simultaneous analysis with the mass cytometer (Helios-Hyperion, Fluidigm), according to the manufacturer’s instructions. The metal isotopes associated with each region of interest were simultaneously measured, and yielded an intensity map of the target proteins throughout each spot.

MCD Viewer v1.0.560.6 (Fluidigm) was used to visualize images, and cell segmentation masks were generated by ilastik Version 1.3.2. Subsequently, the cell segmentation masks were imported into Cell Profiler Version 3.1.5 to extract single-cell information. The single-cell data was then normalized to the 99^th^ percentile for phenograph clustering according to an algorithm implemented in histoCAT (Version 1.76). T-SNE dimensionality reduction, PhenoGraph cluster plots and neighborhood analyses were generated by histoCAT. Absolute quantification of α-SMA^+^ CAFs and immune cells was performed using 5 different pixel (1 μm^2^/pixel) area with the highest infiltration strength.

### Multiplex immunofluorescence staining

We performed multiplex immunofluorescence staining of 4 μm formalin-fixed, paraffin-embedded sections by using a PANO 7-plex IHC kit (Panovue cat 0004100100, Beijing, China) according to the manufacturer’s instructions, as previously described [12]. The different primary antibodies used were: α-SMA (1:50, ab7817, Abcam), CD163 (1:300, #93498, CST), CD4 (1:200, BX22300130, Biolynx), CD8A (1:300, #70306, CST), CTLA4 (1:100, ab237712, Abcam), and FOXP3 (1:50, BLG320202, Biolegend).

Multispectral images were obtained by using the Polaris System (PerkinElmer, Massachusetts, USA), which captures fluorescent spectra from 420 to 720 nm with identical exposure time. Multispectral images were further analyzed using Inform advanced image analysis software (PerkinElmer, Massachusetts, USA). We created a spectral library and spectral unmixing algorithm by using unstained and single opal dye-stained images. Then we used this spectral library to reconstruct images and extract targeted cells for subsequent statistical analyses. Five random high-power fields (20X magnification) inside the region of interest were analyzed per sample.

### Patients and samples

For the retrospective study, all patients were recruited between 2012 and 2014 from Shantou Central Hospital. The cases were selected in this study only if patients underwent curative resection with esophageal squamous cell carcinoma (ESCC), a follow-up was obtained and clinical data were available. The patients who suffered from severe post-operative complications and those who died of other tumors or other causes were excluded. In total, formalin-fixed, paraffin-embedded tumor tissue specimens were obtained from 202 patients, we randomly assigned all participants to a generation dataset (*n* = 103) and validation dataset (*n* = 99). Information about age, gender, therapies and histopathological features was obtained from the medical records and summarized in Supplementary Table S3. Overall survival (OS) was defined as the interval between surgery and death from cancer, or between surgery and the last observation taken for surviving patients. Disease-free survival (DFS) was defined as the interval between surgery and diagnosis of relapse or death. Ethical approval was obtained from the ethical committee of Shantou Central Hospital and the ethical committee of Shantou University Medical College. Only resected samples from surgical patients with written informed consent were included in this study.

### Immunohistochemical (IHC) analysis

IHC staining was performed using a PV-9000 two-step Polymer Detection System (ZSGB-BIO, Beijing, China) and a Liquid DAB Substrate kit (Invitrogen, San Francisco, CA) according to the manufacturer’s instructions, as described previously [13]. Mouse anti-α-SMA monoclonal antibody (1:150, ab8717, Abcam), and ready-to-use CD68 (ZM-0464) and CD163 (ZM-0428) from ZSGB-BIO (Beijing, China) were used in the IHC analysis.

Each section was independently scored by two histopathologists (He J.Z. and Wang S.H.) without knowing the clinical information of the patients, and discrepancies were resolved by consensus. The staining scores for α-SMA^+^ fibroblasts were analyzed in the lamina propria, stroma, invasive front and the leading edge of ESCC tumors. Each location was scored based on the intensity and area of positive staining. Intensity grade was scored as follows: 0, negative; 1, weak staining; 2, moderate staining; and 3, strong staining. The percentage of positive cells was scored as follows: 0, 0–5%; 1, 6%-25%; 2, 26%-50%; 3, 51%-75%; and 4, > 75%. The final score was achieved by multiplying the intensity and the percentage of positive cells producing a total range of 0 to 12.

Carcinoma nest and stromal areas were applied for quantifying the density of CD68^+^ MØs, and CD163^+^ MØs by an automated quantitative pathology imaging system (Perkin Elmer, Waltham, MA, USA). As shown in Supplementary Fig. S1, we used Vectra 2.0.8 for automated image acquisition (more than 20 most representative fields were selected at × 200) and color image generation. The spectral libraries were built by Nuance 3.0 software, and then the color images were evaluated by Inform 1.2 software following three steps: i) segmented tumor region from the tissue compartments; ii) segmented cells from the tumor region; and iii) calculated H score (= (% at 0)*0 + (% at 1 +)*1 + (% at 2 +)*2 + (% at 3 +)*3) based on the optical density, as described previously [14]. Finally, the numbers of positive cells (= total cells*(% at 1 + % at 2 + % at 3)) and corresponding area (0.49 μm^2^/pixel) in each image of every specimen was calculated manually, and the sum of the top 20 values was considered as the count of each sample. For statistical analysis, we had a score for each protein, and positive cells were grouped into two subgroups (high and low) based on X-tile software [15].

### Construction of a survival predictive model according to the stromal signature

First, we used univariate Cox proportional-hazards regression analysis to evaluate the correlation between survival and each stromal signature. Subsequently, we constructed a predictive model by the summation of the expression of each biomarker (high = 1, low = 0) multiplied by its regression coefficient, as described in the equation: $Y\;=\;\left(\beta1\right)\;\times\;\alpha-SMA_{\left(invasive\;front\right)}\;+\left(\beta2\right)\;\times CD{163}_{stroma}\;+\;\left(\beta3\right)\;\times\;pTNM$ [16]. Patients were then divided into three groups (high-risk, medium-risk and low-risk) by the cut-off values generated by X-tile software.

### Statistical analysis

SPSS 19.0 software (SPSS, Chicago, IL) was used for statistical analyses. Differences between areas were assessed by the paired sample t-test and non-parametric Mann–Whitney test. Trends in survival were calculated by Kaplan–Meier survival analyses with the log-rank test. The association of biomarkers and clinicopathological factors was evaluated by Fisher’s exact test, and non-parametric Spearman correlation analysis was employed to estimate the correlation among all biomarkers. The Cox proportional hazards regression model was used for univariate and multivariate analyses. The predictive value of the parameters was determined by receiver operating characteristic (ROC) curve analysis. For the above comparisons, *P* < 0.05 was considered to indicate statistical significance.

## Results

### Fibroblasts are associated with immunoreactivity in ESCC

**Fig. 1:**
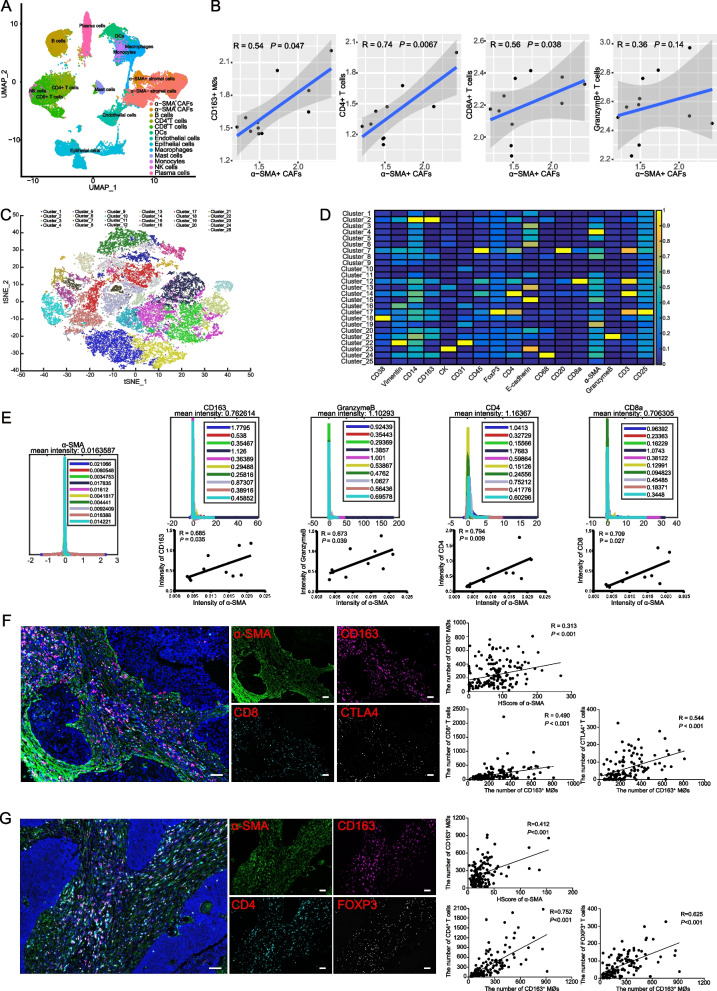
α-SMA^+^ CAFs correlate with immune infiltration in ESCC. **A** UMAP visualization of the 13 distinct cell clusters identified from scRNA-seq data (PRJNA777911) from ESCC tumor samples. **B** Scatter plots showing the correlation between α-SMA^+^ CAFs and immune cell type enrichment score. The relationship was measured by linear regression and non-parametric Spearman correlation. **C** The t-SNE plot combined from 10 ESCC tumor tissues and overlaid with 25 differently colored phenograph clusters, with each color representing one cluster. **D** Heat map of the 25 phenograph clusters were determined by normalized median epitome expression of stained markers using IMC antibodies. **E** The histogram of α-SMA^+^ CAFs, CD163^+^ macrophages (MØs), granzyme B^+^ immune cells, and CD4^+^ and CD8^+^ T cells. Mean intensities for 10 ESCC samples are displayed in different colors. Scatter plots further exhibited the relevance of α-SMA^+^ CAFs to other immune cell types (CD163^+^ MØs, granzyme B^+^ immune cells, CD4^+^ and CD8^+^ T cells) based on the mean intensity. The relation was measured by linear regression and non-parametric Spearman correlation. **F/G** Representative images of multiplex IF staining of ESCC tumor samples. Scale bar = 100 μm. Scatter plots showing the positive correlation between α-SMA^+^ CAFs and CD163^+^ MØs (*n* = 12), or between CD163^+^ MØs and immunologic effector cells (CTLA4^+^, FOXP3^+^, CD4^+^ and CD8^+^ cells) (*n* = 12). *P*-values were determined by non-parametric Spearman correlation analysis

Our recent study uncovered that ESCC is heterogeneous with a highly complex TME composed of fibroblasts and multiple types of immune cells [12]. α-SMA^+^ CAF-derived matrix proteins and cytokines play a vital role in promoting carcinogenesis and tumor progression [9], whereas their complex role in tumor immunization remains poorly understood. Therefore, we performed scRNA-Seq [12] to gain further insight into the relationship between α-SMA^+^ CAFs and immunoreaction. Epithelial cells, fibroblasts (α-SMA^+^ CAFs and α-SMA^−^ fibroblasts), immune cells (macrophages, monocytes, mast, NK, plasma, B and T cells) and endothelial cells were identified based on established markers for known cell types (Fig. 1A). The different cell subsets expressing distinct genes are shown in Fig. S2. Interestingly, we identified a positive correlation between α-SMA^+^ CAF density and the number of M2-like CD163^+^ MØs (*R* = 0.54; *P* = 0.047) that enhance tumor progression, as well as immunologic effector cells that are presumably tumor suppressive, such as CD4^+^ (*R* = 0.74; *P* = 0.0067) and CD8^+^ (*R* = 0.56; *P* = 0.0038) T cells, and a weak correlation with granzyme B^+^ (*R* = 0.36; *P* = 0.14) immune cells (Fig. 1B). In order to further verify this paradoxical result, we performed IMC and multiplex IF staining, which provided highly corroborating evidence at the spatial level in the TME (Fig. 1C-G). Specifically, the abundance of α-SMA^+^ CAFs correlated with CD163^+^ MØs, in addition, we observed significantly positive correlations among α-SMA^+^ CAFs, CD163^+^ MØs and anti-tumor immune cells (i.e. CTLA4, FOXP3, CD4 and CD8 T cells, Fig. 1E-G). These results together indicate that α-SMA^+^ CAFs had an impact on the recruitment of both tumor-promoting and anti-tumor immune cells, and a thorough knowledge of this internal interaction could help identify a crucial target for the treatment of ESCC.

### Invasive front fibroblasts promote disease progression and predict unfavorable survival in ESCC patients

**Fig. 2:**
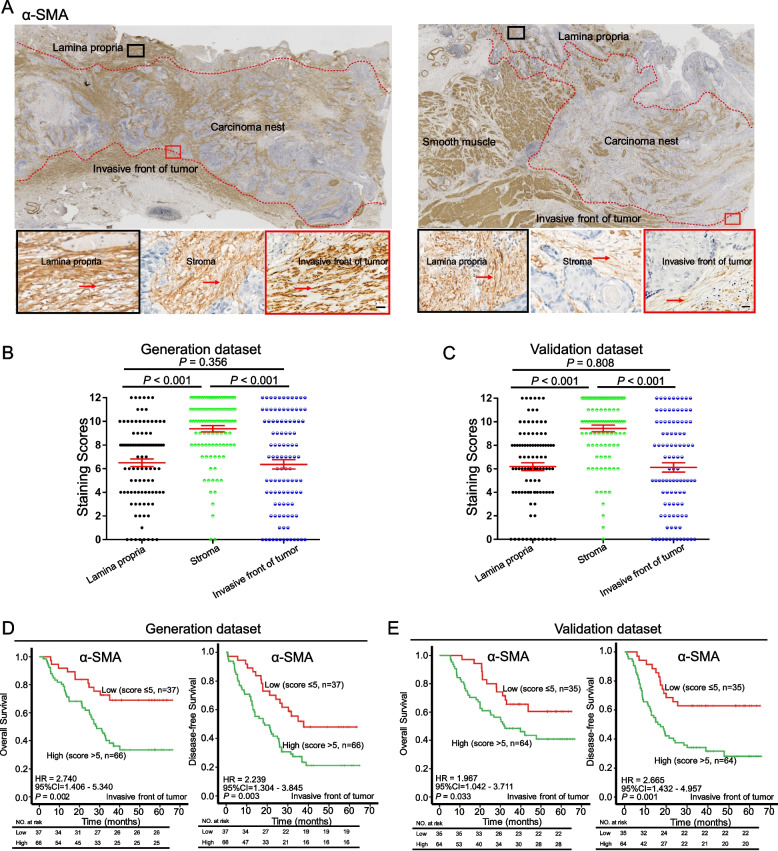
α-SMA^+^ CAFs at the invasive front correlate with clinical outcome of ESCC patients. **A** Representative images of α-SMA IHC staining in ESCC samples (scale bars, 50 μm). **B** IHC scores of α-SMA^+^ CAFs at the invasive front and lamina propria are lower than that in the tumor stroma of ESCC. **C/D** Kaplan–Meier curves indicate that high α-SMA^+^ CAFs at the invasive front of ESCC correlate with shorter OS and DFS in ESCC patients

Given the fundamental roles of CAFs in the TME and the pathobiology of tumors [9], we further interrogated the significance of CAFs in ESCC by employing IHC to detect the expression of α-SMA, a marker for conventional CAFs. As shown in Fig. 2A-B, widespread α-SMA^+^ CAFs were found in both the lamina propria and the tumor stroma, whereas the number of α-SMA^+^ CAFs at the invasive front showed a bipolar distribution in these ESCC samples. Not surprisingly, tumor stroma had the highest number of α-SMA^+^ CAFs compared with the other two regions (Fig. 2A-C). To evaluate the clinical significance of α-SMA^+^ CAFs, Kaplan–Meier survival analysis was performed in two independent cohorts of ESCC patients (generation dataset, *n* = 103; validation dataset, *n* = 99). As shown in Fig. 2D-E, the quantity of α-SMA^+^ CAFs at the invasive front [α-SMA^+^ CAFs_(invasive front)_] was significantly associated with unfavorable prognosis (generation dataset, OS: *P* = 0.002; DFS: *P* = 0.003; validation dataset, OS: *P* = 0.033; DFS: *P* = 0.001). However, as shown in Supplementary Fig. S3, the survival of patients was not statistically different between the high and low groups based on the quantity of α-SMA^+^ CAFs in the lamina propria, or the stroma, or all regions combined (total levels). In addition, high levels of α-SMA^+^ CAFs were closely associated with invasive depth (Supplementary Table S4: generation dataset, *P* < 0.001; validation dataset, *P* = 0.006). Collectively, these data suggest that α-SMA^+^ CAFs at the invasive front may play a crucial part in ESCC progression.

### α-SMA^+^ CAFs at the invasive front are associated with the accumulation of macrophages (MØs) and unfavorable prognosis

**Fig. 3:**
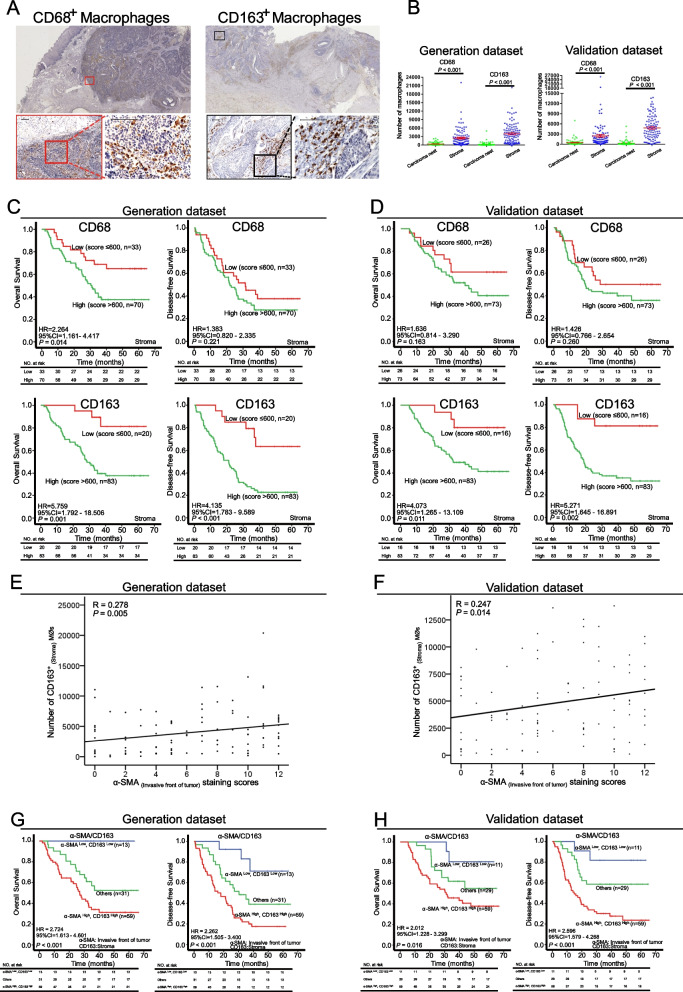
Localization of macrophages in the TME correlates with clinical outcome in ESCC patients. **A** Representative IHC images showing the differential distribution and morphology of CD68^+^ and CD163^+^ macrophages (MØs) in ESCC specimens (scale bars, 50 μm). **B** Comparison of the distribution of CD68^+^ and CD163^+^ MØs in ESCC carcinoma nest and stroma. **C/D** Prognostic values of stromal CD68^+^ and CD163^+^ MØs in ESCC patients in the generation and validation datasets were assessed by Kaplan–Meier survival analysis. **(E/F)** Correlation between α-SMA_(invasive front)_ and stromal CD163^+^ MØs in ESCC patients, as estimated by non-parametric Spearman correlation analysis. **(G/H)** Kaplan–Meier curves showing the prognostic value of a combined index composed of α-SMA^+^ CAFs_(invasive front)_ and stromal CD163^+^ MØs

To examine the contribution of CAFs to immunoreaction, MØ markers, *i.e.* CD68 (pan-MØs) and CD163 (M2 MØs) [17], were detected by IHC in the same cohorts of ESCC samples. As shown in Fig. 3A-B, CD68^+^ MØs were detected in both the peritumoral and intratumoral areas. However, CD163^+^ MØs were distributed mainly in the peritumoral regions and minimally in the intratumoral region. Moreover, compared with the relatively classical morphology of CD68^+^ MØs, *i.e.* mostly flattened and rounded, the morphology of CD163^+^ MØs were pleomorphic, displaying round, oblong, pancake-like, or elongated spindle-like shapes (Fig. 3A). We next analyzed the prognostic value of the two types of MØs. Interestingly, Kaplan–Meier analysis confirmed that the density of intratumoral or peritumoral CD68^+^ MØs could not predict prognosis with certainty (Fig. 3C-D and Fig. S4A-B). In comparison, high levels of CD163^+^ MØs, no matter whether intratumoral or peritumoral localization, strongly correlated with both shorter OS and shorter DFS (Fig. 3C-D and Fig. S4C-D). In view of the tissue heterogeneity, we also normalized the absolute number of CD68^+^ and CD163^+^ MØs with area. Similar to the absolute number, high density of CD163^+^ MØs in the stroma or carcinoma nest were associated with poor outcome in patients with ESCC (Fig. S5A-D). Nevertheless, there were no significant survival differences for both the density of CD68^+^ MØs in the stroma and carcinoma nest (Fig. S5A-D). For the convenience of clinical utilization, we used the absolute number of immune cells for subsequence analysis. To gain insight into how the two types of MØs contribute to the pathogenesis of ESCC, we also analyzed their correlation with the clinical characteristics of ESCC patients. As shown in Supplementary Table S4, no significant correlation was observed for CD68^+^ or CD163^+^ MØs.

Our finding of a positive correlation between α-SMA^+^ CAFs_(invasive front)_ and the accumulation of inflammatory cells prompted us to examine their association with MØs_(stroma)_, also an indicator of inflammation. We found that α-SMA^+^ CAFs_(invasive front)_ were positively correlated with both MØs, especially M2 types of MØs (Fig. 3E-F and Fig. S4E-F). We then asked whether a high density of both α-SMA^+^ CAFs_(invasive front)_ and MØs_(stroma)_ contribute to clinical outcome of ESCC patients. As expected, we found that tumors having high levels of both α-SMA^+^ CAFs_(invasive front)_ and CD163^+^_(stroma)_ (generation dataset, OS: *P* < 0.001; DFS: *P* < 0.001; validation dataset, OS: *P* = 0.016; DFS: *P* < 0.001, Fig. 3G-H) predicted poor outcome. These data suggest that α-SMA^+^ CAFs may enhance the recruitment of inflammatory cells, especially CD163^+^ MØs, to promote disease progression in ESCC patients.

### A barrier consisting α-SMA^+^ CAFs and CD163^+^ MØs at the invasive front blocks tumor-infiltrating immune cells

**Fig. 4:**
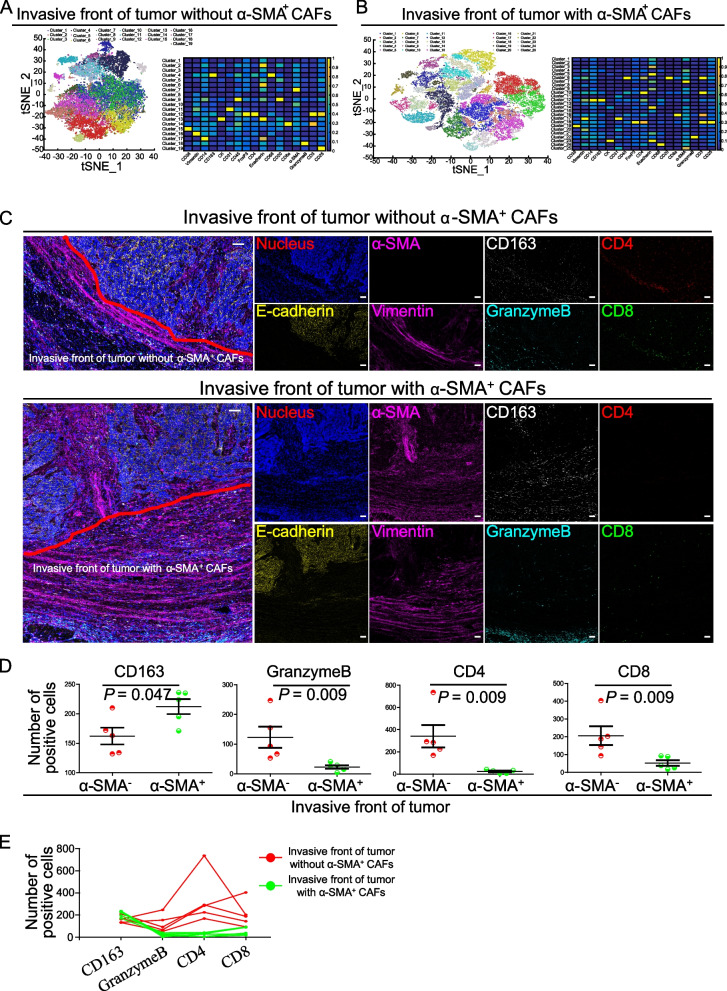
Combination of α-SMA^+^ CAFs and CD163^+^ MØs at the ESCC invasive front prevent immune invasion. **A**/**B** Unsupervised clustering of all cell phenotypes in the IMC data of ESCC invasive front tissue with (*n* = 5) or without (*n* = 5) α-SMA^+^ CAFs based on their biomarker expression. Cell phenotypes were visualized with heatmaps. **C** Representative IMC images of ESCC invasive front tissue with or without α-SMA^+^ CAFs. Each image in the left was rendered with a selection of different markers (blue: nucleus, yellow: epithelial cell, magenta: α-SMA^+^ CAFs or vimentin^+^ fibroblasts, white: CD163^+^ MØs, red: CD4^+^ T cell, green: CD8^+^ T cell, cyan: granzyme B^+^ immune cells). Scale bar = 100 μm. **D** Comparison of tumor infiltrating immunocytes for each cell subtype between the ESCC invasive front tissue with (*n* = 5) or without (*n* = 5) α-SMA^+^ CAFs (non-parametric Mann–Whitney). **E** The trend lines display the patient immune cell subtype pattern of change. Dense presence of CD163^+^ MØs in the ESCC invasive front with α-SMA^+^ CAFs tends to reduce the number of tumor-infiltrating immune cells (granzyme B^+^ immune cells, CD4^+^ and CD8^+^ T cells)

To further explore the inverse association between α-SMA^+^ CAFs and infiltrating immune cells, we performed IMC and reanalyzed the microenvironmental characteristics of tumor specimens with or without α-SMA^+^ CAFs in the leading-edge area. Specific cell types were found to be clustered together in the tissue with α-SMA^+^ CAFs in the leading-edge area, according to clustering analysis and t-SNE visualization, whereas the cell types were mixed in the tissue without α-SMA^+^ CAFs in the leading-edge area (Fig. 4A-B). The histopathological features of tumor tissue with or without α-SMA^+^ CAFs in the leading-edge area were further characterized, and showed that the presence of α-SMA^+^ CAFs at the invasive front form a fibrous barrier with CD163^+^ MØs to hinder immunologic effector cell infiltration (such as granzyme B^+^ immune cells, CD4^+^ and CD8^+^ T cells), whereas the absence of α-SMA^+^ CAFs in the leading-edge area was associated with high intratumoral immune infiltration (Fig. 4C). We quantitatively discovered that, as compared to the group without α-SMA^+^ CAFs, the density of CD163^+^ MØs was increased in the α-SMA^+^ CAF group (*P* = 0.047), and the expression of intratumoral effector immune cell markers granzyme B, CD4 or CD8 showed a dramatic drop (Fig. 4D). In support, a line chart further confirmed that, compared with the invasive front without α-SMA^+^ CAFs, the invasive front of the same patient with α-SMA^+^ CAFs demonstrated a trend with considerably higher infiltrating density of CD163^+^ MØs and a lower level of immune cell counts, including granzyme B^+^ cytotoxic T cells, and CD4^+^ and CD8^+^ T cells (Fig. 4E). Thus, these results suggest that the distribution of immune cells may be influenced by α-SMA^+^ CAFs at the invasive front.

### Spatial interaction and intercellular communication networks of α-SMA^+^ CAFs in the invasive front 

**Fig. 5:**
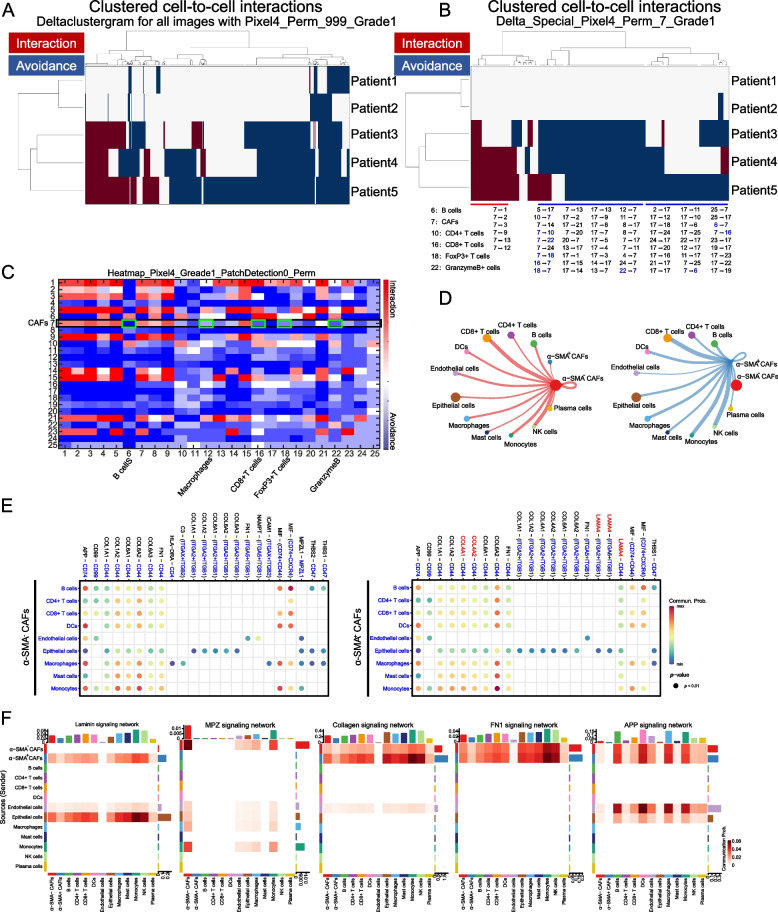
Spatial interaction and intercellular communication networks of α-SMA^+^ CAFs in the ESCC invasive front. **A** Visualization of unsupervised neighborhood analysis for all cell-to-cell interactions in α-SMA^+^ CAFs_(invasive front)_ patient tissues based on the presence of significant neighboring (red) or avoidance (blue), and white represents cell interactions that are not present or not significant (permutation test, *P* < 0.01). **B** Agglomerative clustering of highlighted α-SMA^+^ CAFs (cell phenotype 7), the clustered heatmap revealed that they surrounded multiple cell phenotypes and avoided various immune cell types (such as B cells (phenotype 6), T cells (phenotype 10 and 16), FoxP3^+^ Tregs (phenotype 18), and granzyme B^+^ cells (phenotype 22)). The avoidances for each interaction cluster are listed below the dendrogram. The specific cell types are indicated by numbers displayed in the heatmap of Fig. 4B. Blue highlights the immune cell subtypes avoided with α-SMA^+^ CAFs. **C** Heatmap displaying all interactions in 5 ESCC invasive front tissues with α-SMA^+^ CAFs where the cell phenotype in the row was significantly neighbored (red) or avoided (blue) by the cell type in the column (permutation test, *P* < 0.01). **D** Interaction weight plot of CAFs. The thicker the line, the stronger the interaction weight/strength between the two cell types. **E** Difference of key interacting ligands in the outing and incoming signal mode between α-SMA^+^ CAFs and α-SMA^−^ CAFs, as analyzed by CellChat. **F** Communication probability of a signaling pathway shown in the heatmap. The likelihood of communication between the two cell subtypes increases with color intensity

Through ligand-receptor interactions and extracellular signaling, CAFs have a significant impact on TME remodeling [9, 18]. To explore the cell–cell interactions between CAFs and immune cell subtypes, we applied the spatial neighboring analysis of histoCAT and CellChat methods on ESCC tumor tissues. The interaction and avoidance between or within α-SMA^+^ CAFs and immune cell phenotypes were considerably enriched (Fig. 5A). Among them, α-SMA^+^ CAFs had significantly more interaction with multiple tumor cell subtypes (especially vimentin^+^ α-SMA^+^ E-cadherin^+^ tumor cells, cluster_1-3/9). In contrast, observable avoidance with various immunologic effector cells, including granzyme B^+^ cytotoxic T cells, FoxP3^+^ Tregs, and CD4^+^ and CD8^+^ T cells (Fig. 5B-C), and a weak interaction between α-SMA^+^ CAFs and CD163^+^ MØs was observed (Fig. 5B-C, cluster_12/13). This is consistent with histomorphology in the invasive front TME (Fig. 4C), where tumor cells are surrounded by α-SMA^+^ CAFs, and CD163^+^ MØs hinder intratumoral immune infiltrate. Moreover, the expression of various ligand-receptor pairs was investigated to decipher the cell–cell interactions in the TME with or without α-SMA^+^ CAFs, and display vast communication with nearly all other cell clusters (Fig. 5D and Fig. S6). In contrast with α-SMA^−^ CAFs, the number of interactions (especially between macrophages, monocytes, DCs, B cells, and CD4^+^ and CD8^+^ T cells) was substantially higher in α-SMA^+^ CAF samples (Fig. 5D). CellChat was used to analyze the communication among cells in the TME, and we identified a complex interaction network between CAFs with or without α-SMA and cell clusters. Collagen family genes were highly expressed by α-SMA^+^ CAFs, including collagen type IV alpha 1 chain (COL4A1) and collagen type IV alpha 2 chain (COL4A2)-CD44, which are involved in cell adhesion and migration [19]. Moreover, α-SMA^+^ CAFs highly expressing laminin (LAMA4) may also interact with other immune and epithelial cells though integrins (ITGA2, ITGA6 and ITGB1) and CD44, which mediate cell adhesion and recognition (Fig. 5E) [19]. We also used the “netVisual heatmap” to visualize the details of information flow among the cellular components (Fig. 5F and Fig. S6), and found that the laminin signaling pathway showed a strong inter-association between α-SMA^+^ CAFs and other cells, such as macrophages, monocytes, mast cells and plasma cells (Fig. 5F). In addition, MPZ (myelin protein zero) signaling showed an association between α-SMA^−^ CAFs and macrophages or monocytes. However, the collagen, FN1 and APP signaling networks were generally involved in interaction with α-SMA^+^ CAFs or α-SMA^−^ CAFs in the TME (Fig. 5F). Notably, compared to α-SMA^−^ CAFs, α-SMA^+^ CAFs were not found to interact with other cell types (for instance macrophages, T cells and NK cells) through the CLEC, ICAM and MHC-II signaling pathway (Fig. S7).

In order to further validate the activated or inhibited pathways in α-SMA^+^ CAFs, we performed differentially-expressed gene (DEG) analysis comparing α-SMA^−^ CAFs versus α-SMA^+^ CAFs and found a total of 479 DEGs in the α-SMA^+^ CAFs (Supplementary Table S5). Subsequently, we filtered the DEGs by comparing the percent of α-SMA^−^ CAFs and α-SMA^+^ CAFs that express each gene (Fig. S8A). Of those genes, the significantly downregulated DEGs were related to inflammatory response, i.e. chemokines and chemokine receptors CCL11, CCL20, CXCL1, CXCL3, CXCL5, CXCL6; interleukin IL6, IL1B, IL33, within α-SMA^+^ CAFs (Fig. S8A). By contrast, the upregulated DEGs in α-SMA^+^ CAFs (such as cytokines and their ligands CCL8, EGFL6, IGFBP2, IGFBP7 and TGFB1I1) were connected with the regulation of immune response (Fig. S8A). Indeed, KEGG enrichment analysis suggested that the DEGs affected the VEGF, IL-18 and TGF-β signaling pathways (Fig. S8B). Thus, these results imply different functions of CAFs in ESCC.

### A molecular prognostic model based on stromal signatures

**Fig. 6:**
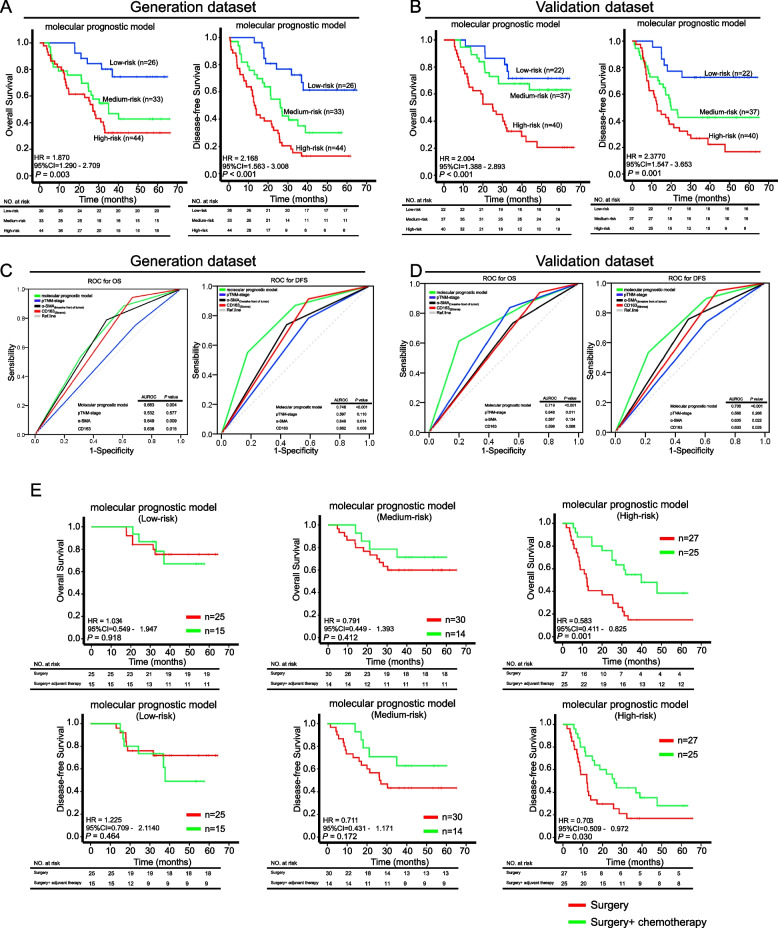
Identification of a novel molecular prognostic model in ESCC patients. **A/B** The new molecular prognostic model was generated based on the expression level and localization of three stromal components, *i.e.* α-SMA^+^ CAFs, CD163^+^ MØs, and pTNM-stage. Patients were divided into low-, medium- and high-risk groups based on X-tile software analysis. Kaplan–Meier graphs showing that patients in the low-, medium-, and high-risk groups had contrasting OS and DFS. **C/D** Receiver operating characteristic (ROC) curves were used to compare the prognostic power of the new molecular prognostic model, each of the stromal components, and pTNM-stage in the generation and validation dataset. **E** The new molecular prognostic model identifies a high-risk group of ESCC patients who can benefit from chemotherapy. Kaplan–Meier survival analysis indicates that chemotherapy prolonged patient OS and DFS in the high-risk group, but not in the low- and medium-risk groups

The intercorrelation and high clinical significance of the stromal components in ESCC prompted us to develop an optimal forecasting model that could be generated based on a specific stromal signature. To this end, we calculated the risk score Y based on the quantity of stromal components that showed significant correlation with prognosis, *i.e.* Y = (β1) * (α-SMA^+^ CAFs_(invasive front)_) + (β2) * (CD163^+^ MØs_(stroma)_) + (β3) * pTNM. In the dataset, β1 = 0.812, β2 = 1.788, and β3 = 1.063, which were regression coefficients that were calculated by univariate Cox proportional hazards analysis. Thus, the Y value represents the summation of the expression of each biomarker (high = 1, low = 0) multiplied by its regression coefficient. Based on the Y scores, the patients were divided into high-, medium-, and low-risk groups according to their prognosis, and the optimal cutoff values were determined by X-tile software [15]. Kaplan–Meier analysis further validated that patients in the high-risk group indeed had markedly decreased OS (generation dataset: *P* = 0.001; validation dataset: *P* < 0.001) and DFS (generation dataset: *P* < 0.001; validation dataset: *P* = 0.001, Fig. 6A-B). The 5-year OS rates for high-, medium-, and low-risk groups were 32.4%, 42.9% and 74.5% in the generation dataset, and 20.6%, 63.1% and 71.6% in the validation dataset, respectively. A similar trend was found for the 5-year DFS rate in those groups (generation dataset: 12.8%, 30.0% and 61.3%; validation dataset: 16.7%, 42.7% and 72.2%, respectively). To further test whether this new prognostic model can be used as an independent predictor for survival, we performed both univariate and multivariate analyses. As shown in Supplementary Table S6, the prognostic model was a strong independent predictor. In addition, receiver operating characteristic (ROC) analysis suggested that the predictive power of this prognostic model was higher than that of each stromal component or pTNM-stage (Fig. 6C-D).

### Potential of the molecular prognostic model in identifying ESCC patients who can benefit from adjuvant therapy

The data shown in Supplementary Table S3 suggests that adjuvant therapy does not significantly improve the survival of patients. Given that CAFs and the TME are important determinants of patient response to therapy [9, 11], we tested whether our molecular prognostic model based on stromal signatures can identify ESCC patients who benefited from adjuvant therapies. Therefore, we combined the patients, who received only surgery and those who had surgery + chemotherapy, in both the generation dataset and validation dataset for subsequent analysis. Interestingly, our molecular prognostic model identified a group of high-risk patients in which chemotherapy significantly improved prognosis (OS: *P* = 0.001, DFS: *P* = 0.030, Fig. 6E). However, no significant association was found for the low- and medium-risk groups (Fig. 6E). These data corroborate that stromal signatures, as a complex whole of the TME, may be potentially used to guide the therapeutic option of using adjuvant therapy in the treatment of ESCC patients.

## Discussion

ESCC is among the deadliest cancers worldwide due to its high aggressiveness and dismal prognosis [6, 7], but the application of molecular signatures for predicting the efficacy of adjuvant treatment and prognosis is still rare clinically. In this study, we explored the clinical value of the distribution and composition of the TME, one of the pivotal components in the tumor that is associated with chemoresistance and tumor progression [9–11]. For the first time, we found that increased numbers of CAFs at the invasive front are correlated with cancer progression and prognosis of ESCC patients. Interestingly, the impact of CAFs in the invasive front are related to the immune cell infiltration in different regions of the tumor, depending on the identity of the immune cell subsets. Our data highlight that 1) the activated fibroblasts may interact with immune cells to form a favorable niche for cancer cells at the invasive front, and 2) the various components of the complex TME are orchestrated into a specific spatial order to coordinate various functions during cancer progression. Importantly, we define that a combination of TME components, comprised of α-SMA^+^ CAFs at the invasive front, CD163^+^ MØs and pTNM, not only shows superior prognostic power than the current pTNM staging system, but also can predict patient sensitivity to chemotherapies.

Fibroblasts are the main cell type of the microenvironment, transform into cancer-associated fibroblasts (CAFs) in the tumor microenvironment and are thought to favor tumor progression [20]. Studies have confirmed that most tumors follow the activation of tumor microenvironment remodeling, such as atypical ductal hyperplasia-associated fibroblasts (AHFs), and that induces malignant cell proliferation, migration and invasion of epithelium-like tumor cells [21–23]. Previous reports have demonstrated that AHFs or CAFs regulate growth and polarity changes of tumor cells through the NF-κB pathway, and differ from normal fibroblasts (NFs) in phenotypic characteristics, expression of growth factors and molecular synthesis of the extracellular matrix (ECM) [23]. CAFs not only play an active role in tumorigenesis and development through soluble factors and direct intercellular contacts, but also shape the tumor microenvironment by directly suppressing anti-tumor immune responses or recruiting immunosuppressive cells, However, AHFs suppress T cell proliferation, similar to NFs [24, 25]. These interactions between CAFs and immune cells are of crucial importance for tumor development and progression [25].

The important role played by CAFs has been documented in both tumor initiation and malignant progression. Previous studies have shown that CAFs promote carcinoma progression by inducing EMT via the IL-6/JAK2/STAT3 pathway or paracrine TGF-β [4, 9, 26]. However, our findings reveal that invasive front CAFs are also closely associated with invasive depth. This data suggests that invasive front CAFs in ESCC may operate through distinct mechanisms to promote tumor invasion. A possible mechanism is that activated CAFs, e.g. by TGF-β, upregulate growth factors and chemokines to promote ECM synthesis and immune cell recruitment, leading to a reconstructed tissue architecture that favors tumor growth and progression [1, 9, 27]. This mechanism has also been shown to trigger tumor initiation [28]. For instance, disruption of the TGF-β signaling by conditional inactivation of the TGFβR2 gene in normal fibroblasts in mice led to intraepithelial neoplasia in prostate and invasive squamous cell carcinoma of the forestomach [29]. The mechanism behind this observation is that normal fibroblasts lacking TGFβR2 undergo myofibroblastic differentiation, recapitulating the tumor inductive activity of CAFs that secret various tumor-promoting factors, such as SDF1/CXCL12, FGF, HGF, and BMPs [9, 30]. Remodeling of the TME by CAFs is important for the formation of a niche for carcinoma cell invasion [3, 28]. In keeping with these findings, CAFs have been shown to generate a favorable TME for ESCC cells to invade, colonize and form metastases in distant organs [5]. Notably, recent studies found that CAFs are not only derived from resting tissue fibroblasts, but also endothelial cells, mesenchymal stem cells, bone marrow-derived precursors, and even cancer cells themselves [1, 31]. Gene expression profiles demonstrate that fibroblasts of distinct anatomical origin have distinct differentiation patterns for distinct functions [1, 9, 32]. High HSF1 expression in CAFs is strongly associated with tumor progression and poor gastric cancer patient outcome [33]. ANGTL4, MMP13 and STC1 secretion by CAFs promotes metastasis of colorectal cancer [34]. Therefore, when analyzing the function and clinical significance of CAFs, as a complex component of the TME, cell origin, distribution, active signal pathways and genome must be taken into consideration.

Cancer progression involves the crosstalk between different cell types within the carcinoma and the stroma [35]. Macrophages (MØs) represent a major type of infiltrating leukocyte that is highly plastic and has contradictory roles in cancer [2]. Indeed, in this study, we show that the clinical significance of peritumoral CD68^+^ MØs and those in the intratumoral region have opposing functions. This result is in keeping with the finding that intratumoral CD68^+^ MØs predict good outcome, but MØs in the peritumoral areas promote tumor cell invasion and metastasis in hepatocellular carcinoma [36, 37]. Classically activated M1 macrophages (M1 MØs) participate in antigen presentation, promote T helper 1 (Th1) immune responses and suppress tumor initiation [2, 38]. In contrast, alternatively activated M2 macrophages (M2 MØs) facilitate Th2 immune responses, play anti-inflammatory roles, and promote carcinoma progression [2, 38]. However, the mechanisms underlying how macrophages initially transform from anti-tumor properties to pro-tumor properties are not fully understood. It has been shown that CAFs play a pivotal role in converting M1 MØs to M2 MØs in the TME. For instance, IL-10 and TGF-β1 derived from CAFs up-regulate the expression of CD163 in monocytes in oral squamous cell carcinoma [39]. CAF-derived IL-6 upregulates the expression of macrophage colony-stimulating factor (M-CSF) in monocytes, which switches monocyte differentiation to M2 MØs rather than dendritic cells, eventually leading to the suppression of T cell activation [40, 41]. More recent studies revealed that CAFs release chemokines to recruit MØs to the tumor, and the infiltration of CAFs are associated with the number of CD68^+^ or CD163^+^ MØs in patients with oral squamous cell carcinoma [39, 42]. These findings are in agreement with our data, which show that invasive front CAFs are positively associated with the accumulation of CD163^+^ MØs. More importantly, we found that the concurrent presence of high-level CAFs and MØs significantly correlates with shorter patient survival in ESCC. Collectively, our data indicate that CAFs interact with MØs and work in concert in shaping the TME. Nevertheless, the molecular mechanisms remain unclear and warrant further investigation.

According to the anti-tumor immune response state, cancers can be categorized into three phenotypes: inflamed, immune-excluded and immune desert [43]. The immune-excluded phenotype is characterized by immune cells that have been retained in the stroma that surround carcinoma nests [43]. In agreement with our data, a complete barrier in an invasive front consisting of α-SMA^+^ CAFs and CD163^+^ MØs can prevent immune cells from infiltrating tumors, such as granzyme B^+^ cytotoxic T cells, FoxP3^+^ Tregs, and CD4^+^ and CD8^+^ T cells. These results are supported by recent research in hepatocellular carcinoma [44]. In brief, CAFs may be involved in this process through several mechanisms. Firstly, CAF interaction with MØs in the peritumoral stroma can facilitate the formation of a physical barrier consisting of extracellular matrix components, such as collagen, hyaluronic acid, and fibronectin. To promote collagen deposition, MØs not only secrete lysyl oxidase to induce cross-linking of collagen fibers, but also express Fas-L and release active soluble Fas-L that induces Fas^+^ lymphocyte apoptosis, similar to tumor cells [45, 46]. Moreover, CAFs present Fas-L and PD-L2 via MHC-1 antigen cross-presentation to further suppress the activity of CD8^+^ T cells, resulting in reduced lymphocyte infiltration of the TME [45, 46]. Additionally, hyaluronic acid prompts macrophages to differentiate into an M2 phenotype through miR935-mediated inhibition of C/EBPβ expression, thereby facilitating immune evasion by malignant cells in the TME [45, 46]. The different signaling networks and associated ligand/receptor pairs between two distinct CAFs (with or without α-SMA) and other cell types are also demonstrated by our work, which reinforce the crucial role of fibroblast subsets for both spatial distribution variation and intratumor heterogeneity. Furthermore, the increasing numbers of CAFs at the invasive front are related to the disease progression and prognosis of ESCC patients in our results. This finding suggests that targeting CAFs may have potential therapeutic effects in the treatment of ESCC. Generally, CAF targeting in cancer therapy includes CAF depletion, inhibition of CAF activation, development of CAF-targeted vaccines and therapeutics (i.e., antibody–drug conjugates and bispecific T cell engager (BiTE) technology), and targeting CAF-produced ECM proteins [47]. In the case of CAF-targeted vaccines, for example, the therapeutic strategy of CAFs-targeting vaccines relies on surface markers of CAFs, such as FAP, α-SMA, and PDGFR [47]. Depletion of these markers may be beneficial, as FAPα-expressing vaccines reduce tumor growth by generating a FAPα-specific CTL response capable of killing CAFs. In addition, the reduction of FAPα-expressing CAFs significantly attenuates the expression of collagen I and various stromal factors that contribute to tumor progression [48]. Inhibition of tumor angiogenesis and subsequent suppression of mouse pancreatic tumors was also observed after removal of FAP^+^ CAFs in FAP-targeted chimeric antigen receptor (CAR) T cells [49]. Therefore, breaking down the barrier at the invasive front, resulting in more effective tumor-infiltrating immune cells, may be an effective way to improve the prognosis of patients bearing immuno-excluded tumors by taking into account the spatial distribution and interaction of cell subtypes as a whole.

It has been increasingly recognized that the TME has a dramatic influence on the efficacy of cancer therapies. Previous studies have identified that soluble factors produced by CAFs, such as TGF-β, HGF and IL-6, are important mediators of resistance to anticancer drugs [1, 9]. Other studies suggest that CAFs modulate resistance to immunotherapy by suppressing the activation of T cells via upregulating the expression of PD-L1 and PD-L2 [50, 51]. In addition, it has been proposed that MØs may serve as promising targets for clinical immunotherapy due to their effects on multifarious aspects of tumor progression [52]. More importantly, our findings demonstrate that a molecular prognostic model based on stromal signatures can identify a subgroup of ESCC patients that are responsive to adjuvant treatment (Fig. 6). Taken together, these data suggest that we should comprehensively evaluate the TME to tailor therapeutic strategies, instead of simply targeting a single stromal component.

## Conclusions

To summarize, we have characterized the stromal features of ESCC, and our findings suggest that the localization/distribution of stromal components is one of the determinants of patient prognosis in ESCC. We highlight that invasive front CAFs are associated with unfavorable patient survival and the infiltration of various types of immune cells. Based on our newly characterized stromal signatures in the complex TME in ESCC, we have constructed a prognostic model that can more precisely predict clinical outcome in ESCC patients. This new prognostic model may facilitate the selection of optimal adjuvant therapeutic strategies for ESCC treatment, which may also provide a useful framework for future studies in other cancers.

## Supplementary Information

**Additional file 1:** **Fig. S1.** Representative images showing the scoring process by the automated quantitative pathology imaging system. **Fig. S2.** Violin plots displaying the expression level of representative markers in each cell cluster. **Fig. S3.** Kaplan-Meier survival curves for total α-SMA^+^ CAFs, lamina propria α-SMA^+^ CAFs and stromal α-SMA^+^ CAFs in the generation (*n*=103) and validation (*n*=99) dataset of patients with ESCC. **Fig. S4.** The number of intratumoral macrophages correlates with clinical outcome in ESCC patients. **Fig. S5.** The density of CD68^+^ and CD163^+^ MØs correlates with clinical outcome in patients with ESCC. **Fig. S6.** Crucial cell-to-cell interaction pathways among the distinct cell populations predicted by CellChat. **Fig. S7.** Cell-to-cell communication among the CAFs and other cell types. **Fig. S8.** Differentially-expressed gene (DEG) enrichment analysis for α-SMA^+^ CAFs. **Supplementary Table S1.** The clinicopathological parameters of 11 patients profiled by scRNA-seq. **Supplementary Table S2.** Metal-conjugated antibodies and element-containing reagents used for IMC. **Supplementary Table S3.** Clinicopathological characteristics in the generation and validation dataset of patients with ESCC. **Supplementary Table S4.** Correlation between markers and clinicopathological characteristics in the generation and validation datasets. **Supplementary Table S5.** Differential expressed genes between α-SMA^+^ CAFs and α-SMA^-^ CAFs. **Supplementary Table S6.** Univariate and multivariate analyses of factors associated with overall survival (OS) and disease-free survival (DFS) in the generation and validation datasets of patients with ESCC.

## Data Availability

All data analyzed in this study are available from the corresponding author upon reasonable request.

## Abbreviations

TME: Tumor microenvironment
ESCC: Esophageal squamous cell carcinoma
IMC: Imaging mass cytometry
MØs: Macrophages
pTNM: Pathological tumor-node-metastasis
CAFs: Carcinoma-associated fibroblasts
ECM: Extracellular matrix
α-SMA: α-Smooth muscle actin
COL4A1: Collagen type IV alpha 1 chain
COL4A2: Collagen type IV alpha 2 chain
MPZ: Myelin protein zero
OS: Overall survival
DFS: Disease-free survival
ROC: Receiver operating characteristic
