# Supplementary material for: Spatial analysis of stromal signatures identifies invasive front carcinoma-associated fibroblasts as suppressors of anti-tumor immune response in esophageal cancer
Source: J Exp Clin Cancer Res. 2023 May 31;42:136. doi: 10.1186/s13046-023-02697-y (PMC10230698; doi:10.1186/s13046-023-02697-y)
Supplement: Supplementary file 1 — Additional file 1: Fig. S1. Representative images showing the scoring process by the automated quantitative pathology imaging system. Fig. S2. Violin plots displaying the expression level of representative markers in each cell cluster. Fig. S3. Kaplan-Meier survival curves for total α-SMA+ CAFs, lamina propria α-SMA+ CAFs and stromal α-SMA+ CAFs in the generation (n=103) and validation (n=99) dataset of patients with ESCC. Fig. S4. The number of intratumoral macrophages correlates with clinical outcome in ESCC patients. Fig. S5. The density of CD68+ and CD163+ MØs correlates with clinical outcome in patients with ESCC. Fig. S6. Crucial cell-to-cell interaction pathways among the distinct cell populations predicted by CellChat. Fig. S7. Cell-to-cell communication among the CAFs and other cell types. Fig. S8. Differentially-expressed gene (DEG) enrichment analysis for α-SMA+ CAFs. Supplementary Table S1. The clinicopathological parameters of 11 patients profiled by scRNA-seq. Supplementary Table S2. Metal-conjugated antibodies and element-containing reagents used for IMC. Supplementary Table S3. Clinicopathological characteristics in the generation and validation dataset of patients with ESCC. Supplementary Table S4. Correlation between markers and clinicopathological characteristics in the generation and validation datasets. Supplementary Table S5. Differential expressed genes between α-SMA+ CAFs and α-SMA- CAFs. Supplementary Table S6. Univariate and multivariate analyses of factors associated with overall survival (OS) and disease-free survival (DFS) in the generation and validation datasets of patients with ESCC. [file 13046_2023_2697_MOESM1_ESM.docx]

**Spatial analysis of stromal signatures identifies invasive front carcinoma-associated fibroblasts as suppressors of anti-tumor immune response in esophageal cancer**

Jian-Zhong He PhD^1,2#^, Yang Chen PhD ^1,3^^#^, Fa-Min Zeng PhD^2#^, Qing-Feng Huang PhD ^1,4#^, Hai-Feng Zhang PhD^5^, Shi-Ping Xian MS^2^, Lei Tang MS^2^, Shao-Hong Wang MD^6^, Shuai-Xia Yu MS^1,4^, Xiao-Xiao Pang MS^1,4^, Ye Liu MD^2^, Xiu-E Xu BSc^1,4^, Jian-Yi Wu BSc^1,7^, Wen-Jun Shen PhD^1,8^*, Zhan-Yu Li MS^2^*, En-Min Li PhD^1,7^*, Li-Yan Xu PhD^1,4^*

**Supplementary Files:**

**Fig. S1.** **Representative images showing the scoring process by the automated quantitative pathology imaging system.**

**Fig. S2**. **Violin plots displaying the expression level of representative markers in each cell cluster.**

**Fig. S3**. **Kaplan-Meier survival curves for total α-SMA^+^ CAFs, lamina propria α-SMA^+^ CAFs and stromal α-SMA^+^ CAFs in the generation (n=103) and validation (n=99) dataset of patients with ESCC.**

**Fig. S4. The number of intratumoral macrophages correlates with clinical outcome in ESCC patients.**

**Fig. S5**. **The density of CD68^+^ and CD163^+^ MØs correlates with clinical outcome in patients with ESCC.**

**Fig. S6**. **Crucial cell-to-cell interaction pathways among the distinct cell populations predicted by CellChat.**

**Fig. S7**. **Cell-to-cell communication among the CAFs and other cell types.**

**Fig. S8**. **Differentially-expressed gene (DEG) enrichment analysis for α-SMA^+^ CAFs.**

| **Supplementary Table S1. The clinicopathological parameters of 11 patients profiled by scRNA-seq** | | | | | | | |
| --- | --- | --- | --- | --- | --- | --- | --- |
| **Patient ID** | **Sex** | **Age** | **Histology** | **Stage** | **Smoker** | **Drinker** | **Family history** |
| 787 | Male | 71 | squamous cell carcinoma | Ⅱb | Yes | Yes | No |
| 865 | Male | 57 | squamous cell carcinoma | Ⅲc | Yes | Yes | No |
| 944 | Male | 51 | squamous cell carcinoma | Ⅲc | Yes | Yes | No |
| 986 | Male | 68 | squamous cell carcinoma | Ⅰb | No | No | Yes |
| 658 | Female | 80 | squamous cell carcinoma | Ⅱb | No | No | No |
| 846 | Male | 65 | squamous cell carcinoma | Ⅲa | Yes | Yes | No |
| 009 | Male | 67 | squamous cell carcinoma | 0 | Yes | No | No |
| 836 | Male | 76 | squamous cell carcinoma | Ⅰa | No | No | No |
| 686 | Male | 65 | squamous cell carcinoma | Ⅱb | Yes | No | No |
| 866 | Female | 68 | squamous cell carcinoma | Ⅲc | No | No | Yes |
| 585 | Male | 67 | squamous cell carcinoma | Ⅲa | Yes | No | No |

| **Supplementary Table S2. Metal-conjugated antibodies and element-containing reagents used for IMC** | | | | | |
| --- | --- | --- | --- | --- | --- |
| Antigen | Clone | Metal | Dilution | Supplier | Identifier |
| Vimentin | D21H3 | 143Nd | 1/200 | Fluidigm | 3143027D |
| CD38 | EPR4106 | 141Pr | 1/100 | Fluidigm | 3141018D |
| HistoneH3 | D1H2 | 176Yb | 1/100 | Fluidigm | 3176016A |
| CD20 | H1 | 161Dy | 1/100 | Fluidigm | 3161029D |
| CD3 | Polyclonal | 170Er | 1/100 | Fluidigm | 3170019D |
| CD4 | EPR6855 | 156Gd | 1/100 | Fluidigm | 3156033D |
| CD68 | KP1 | 159Tb | 1/100 | Fluidigm | 3159035D |
| CD8a | D8A8Y | 162Dy | 1/100 | Fluidigm | 3162034D |
| FoxP3 | 236A/E7 | 155Gd | 1/100 | Fluidigm | 3155016D |
| Pan-keratin | C11 | 148Nd | 5ug/ml | Fluidigm | 3148020D |
| Granzyme B | EPR20129-217 | 167Er | 1/200 | Fluidigm | 3167021D |
| Collagen type I | Polyclonal | 169Tm | 1/200 | Fluidigm | 3169023D |
| E-cadherin | 24E10 | 158Gd | 5ug/ml | Fluidigm | 3158021A |
| CD25 | EPR6452 | 175Lu | 1/200 | Fluidigm | 3175036D |
| CD45 | 2B11 | 152Sm | 1/300 | Fluidigm | 3152016D |
| CD163 | EDHu-1 | 147Sm | 1/100 | Fluidigm | 3147021D |
| CD14 | EPR3653 | 144Nd | 1/200 | Fluidigm | 3144025D |
| CD31 | EPR3094 | 151Eu | 1/100 | Fluidigm | 3151025D |
| α-SMA |  | 165Ho | 1/1600 | Fluidigm |  |
| DNA Intercalator | NA | 191Ir, 193Ir | 1/400 | Fluidigm | 201192A |

| **Supplementary Table S3. Clinicopathological characteristics in the generation and validation dataset of patients with ESCC** | | | | | | | | | | | | | |
| --- | --- | --- | --- | --- | --- | --- | --- | --- | --- | --- | --- | --- | --- |
| **Clinical and pathological indexes** | **Generation dataset** | | | | | |  | **Validation dataset** | | | | | |
|  | No. | 5-year OS (%) | *P** |  | 5-year DFS (%) | *P** |  | No. | 5-year OS (%) | *P** |  | 5-year DFS (%) | *P** |
| Specimens | 103 |  |  |  |  |  |  | 99 |  |  |  |  |  |
| Mean age | 58.6 |  |  |  |  |  |  | 57.0 |  |  |  |  |  |
| Age (years) |  |  |  |  |  |  |  |  |  |  |  |  |  |
| <58 | 48 | 42.7 | 0.624 |  | 28.4 | 0.607 |  | 50 | 47.7 | 0.982 |  | 41.1 | 0.951 |
| ≥58 | 55 | 49.6 |  |  | 32.9 |  |  | 49 | 47.7 |  |  | 40.2 |  |
| Gender |  |  |  |  |  |  |  |  |  |  |  |  |  |
| Male | 82 | 49.8 | 0.436 |  | 32.4 | 0.978 |  | 76 | 44.3 | 0.278 |  | 37.5 | 0.403 |
| Female | 21 | 36.4 |  |  | 26.5 |  |  | 23 | 60.2 |  |  | 52.2 |  |
| Therapies |  |  |  |  |  |  |  |  |  |  |  |  |  |
| Only Surgery | 48 | 46.8 | 0.327 |  | 40.4 | 0.360 |  | 34 | 52.9 | 0.980 |  | 46.9 | 0.137 |
| Surgery+chemotherapy | 8 | 25.0 |  |  | 25.0 |  |  | 15 | 44.4 |  |  | 20.0 |  |
| Surgery+ chemoradiotherapy | 47 | 50.7 |  |  | 22.1 |  |  | 50 | 46.5 |  |  | 41.0 |  |
|  |  |  |  |  |  |  |  |  |  |  |  |  |  |
| Tumor size |  |  |  |  |  |  |  |  |  |  |  |  |  |
| ≤3cm | 35 | 56.1 | 0.294 |  | 34.1 | 0.746 |  | 31 | 57.7 | 0.237 |  | 58.1 | 0.202 |
| 3-5cm | 43 | 41.9 |  |  | 28.3 |  |  | 49 | 45.5 |  |  | 34.9 |  |
| >5cm | 25 | 41.2 |  |  | 30.9 |  |  | 19 | 37.6 |  |  | 27.6 |  |
| Tumor location |  |  |  |  |  |  |  |  |  |  |  |  |  |
| upper | 6 | 83.3 | 0.252 |  | 0 | 0.263 |  | 9 | 55.6 | 0.626 |  | 33.3 | 0.251 |
| middle | 55 | 45.4 |  |  | 33.9 |  |  | 51 | 51.5 |  |  | 49.6 |  |
| lower | 42 | 41.4 |  |  | 30.3 |  |  | 39 | 41.5 |  |  | 31.4 |  |
| Histologic grade |  |  |  |  |  |  |  |  |  |  |  |  |  |
| G1 | 20 | 55.0 | 0.436 |  | 44.4 | 0.105 |  | 21 | 61.9 | 0.033 |  | 28.6 | 0.001 |
| G2 | 74 | 44.9 |  |  | 29.0 |  |  | 62 | 51.0 |  |  | 52.0 |  |
| G3 | 9 | 41.7 |  |  | 0 |  |  | 16 | 18.8 |  |  | 12.5 |  |
| Invasive depth |  |  |  |  |  |  |  |  |  |  |  |  |  |
| T1 | 8 | 87.5 | 0.051 |  | 75.0 | 0.069 |  | 7 | 57.1 | 0.544 |  | 57.1 | 0.146 |
| T2 | 19 | 54.1 |  |  | 29.8 |  |  | 16 | 62.5 |  |  | 62.5 |  |
| T3 | 76 | 39.7 |  |  | 25.3 |  |  | 76 | 44.2 |  |  | 34.9 |  |
| Lymph node metastasis |  |  |  |  |  |  |  |  |  |  |  |  |  |
| N0 | 51 | 48.9 | 0.805 |  | 37.1 | 0.206 |  | 50 | 72.0 | <0.001 |  | 60.0 | <0.001 |
| N1 | 32 | 44.1 |  |  | 27.7 |  |  | 22 | 41.3 |  |  | 36.5 |  |
| N2 | 13 | 50.5 |  |  | 0 |  |  | 17 | 11.8 |  |  | 5.9 |  |
| N3 | 7 | 23.8 |  |  | 21.4 |  |  | 10 | 10.0 |  |  | 12.5 |  |
| pTNM-stage |  |  |  |  |  |  |  |  |  |  |  |  |  |
| I | 11 | 63.6 | 0.521 |  | 63.6 | 0.053 |  | 10 | 70.0 | <0.001 |  | 50.0 | <0.001 |
| II | 50 | 43.9 |  |  | 26.7 |  |  | 45 | 73.6 |  |  | 64.4 |  |
| III | 42 | 43.9 |  |  | 35.6 |  |  | 44 | 18.8 |  |  | 15.6 |  |
| *Log-rank test of the Kaplan Meier method; *P* <0.05 was considered to indicate statistical significance. | | | | | | | | | | | | | |
| All patients underwent surgical treatment. | | | | | | | | | | | | | |
| OS: overall survival | | | | | | | | | | | | | |
| DFS: disease-free survival | | | | | | | | | | | | | |

| **Supplementary Table S4. Correlation between markers and clinicopathological characteristics in the generation and validation datasets** | | | | | | | | | | | |
| --- | --- | --- | --- | --- | --- | --- | --- | --- | --- | --- | --- |
| **Variables** | α-SMA^a^ | | *P** |  | CD68^b^ | | *P** |  | CD163^c^ | | *P** |
|  | Low | High |  |  | Low | High |  |  | Low | High |  |
| **Generation dataset** | | | | | | | | | | | |
| Age (years) | | | | | | | | | | | |
| <58 | 15 | 33 | 0.413 |  | 17 | 31 | 0.531 |  | 12 | 36 | 0.217 |
| ≥58 | 22 | 33 |  |  | 16 | 39 |  |  | 8 | 47 |  |
| Gender | | | | | | | | | | | |
| Male | 28 | 54 | 0.458 |  | 9 | 12 | 0.296 |  | 4 | 17 | 1.000 |
| Female | 9 | 12 |  |  | 24 | 58 |  |  | 16 | 66 |  |
| Tumor size | | | | | | | | | | | |
| ≤3 cm | 16 | 19 | 0.049 |  | 11 | 24 | 0.882 |  | 10 | 25 | 0.137 |
| 3-5 cm | 17 | 26 |  |  | 13 | 30 |  |  | 8 | 35 |  |
| >5 cm | 4 | 21 |  |  | 9 | 16 |  |  | 2 | 23 |  |
| Tumor location | | | | | | | | | | | |
| Upper | 2 | 4 | 0.400 |  | 4 | 2 | 0.170 |  | 1 | 5 | 0.244 |
| Middle | 23 | 32 |  |  | 16 | 39 |  |  | 14 | 41 |  |
| Lower | 12 | 30 |  |  | 13 | 29 |  |  | 5 | 37 |  |
| Histologic grade | | | | | | | | | | | |
| G1 | 7 | 13 | 0.856 |  | 7 | 13 | 0.098 |  | 4 | 16 | 0.970 |
| G2 | 26 | 48 |  |  | 26 | 48 |  |  | 14 | 60 |  |
| G3 | 4 | 5 |  |  | 0 | 9 |  |  | 2 | 7 |  |
| Invasive depth | | | | | | | | | | | |
| T1+T2 | 18 | 9 | <0.001 |  | 11 | 16 | 0.337 |  | 8 | 19 | 0.156 |
| T3+T4 | 19 | 57 |  |  | 22 | 54 |  |  | 12 | 64 |  |
| Lymph node metastasis | | | | | | | | | | | |
| N0 | 20 | 31 | 0.542 |  | 18 | 33 | 0.531 |  | 11 | 40 | 0.626 |
| N1+N2+N3 | 17 | 35 |  |  | 15 | 37 |  |  | 9 | 43 |  |
| pTNM-stage | | | | | | | | | | | |
| I | 6 | 5 | 0.349 |  | 5 | 6 | 0.565 |  | 4 | 7 | 0.255 |
| II | 18 | 32 |  |  | 16 | 34 |  |  | 10 | 40 |  |
| III | 13 | 29 |  |  | 12 | 30 |  |  | 6 | 36 |  |
| **Validation dataset** | | | | | | | | | | | |
| Age (years) | | | | | | | | | | | |
| <58 | 18 | 32 | 1.000 |  | 16 | 34 | 0.254 |  | 10 | 40 | 0.414 |
| ≥58 | 17 | 32 |  |  | 10 | 39 |  |  | 6 | 43 |  |
| Gender | | | | | | | | | | | |
| Male | 8 | 15 | 1.000 |  | 7 | 16 | 0.599 |  | 5 | 18 | 0.518 |
| Female | 27 | 49 |  |  | 19 | 57 |  |  | 11 | 65 |  |
| Tumor size | | | | | | | | | | | |
| ≤3 cm | 15 | 16 | 0.125 |  | 7 | 24 | 0.218 |  | 9 | 22 | 0.063 |
| 3-5 cm | 16 | 33 |  |  | 11 | 38 |  |  | 5 | 44 |  |
| >5 cm | 4 | 15 |  |  | 8 | 11 |  |  | 2 | 17 |  |
| Tumor location | | | | | | | | | | | |
| upper | 2 | 7 | 0.110 |  | 0 | 9 | 0.020 |  | 0 | 9 | 0.092 |
| middle | 23 | 28 |  |  | 19 | 32 |  |  | 12 | 39 |  |
| lower | 10 | 29 |  |  | 7 | 32 |  |  | 4 | 35 |  |
| Histologic grade |  |  |  |  |  |  |  |  |  |  |  |
| G1 | 5 | 16 | 0.406 |  | 7 | 14 | 0.707 |  | 2 | 19 | 0.520 |
| G2 | 23 | 39 |  |  | 15 | 47 |  |  | 12 | 50 |  |
| G3 | 7 | 9 |  |  | 4 | 12 |  |  | 2 | 14 |  |
| Invasive depth |  |  |  |  |  |  |  |  |  |  |  |
| T1+T2 | 14 | 9 | 0.006 |  | 7 | 16 | 0.599 |  | 7 | 16 | 0.050 |
| T3+T4 | 21 | 55 |  |  | 19 | 57 |  |  | 9 | 67 |  |
| Lymph node metastasis | | | | | | | | | | | |
| N0 | 23 | 27 | 0.035 |  | 12 | 38 | 0.653 |  | 10 | 40 | 0.414 |
| N1+N2+N3 | 12 | 37 |  |  | 14 | 35 |  |  | 6 | 43 |  |
| pTNM-stage | | | | | | | | | | | |
| I | 4 | 6 | 0.151 |  | 2 | 8 | 0.892 |  | 3 | 7 | 0.325 |
| II | 20 | 25 |  |  | 12 | 33 |  |  | 8 | 37 |  |
| III | 11 | 33 |  |  | 12 | 32 |  |  | 5 | 39 |  |
| *Fisher's Exact Test; *P* <0.05 was considered to indicate statistical significance. | | | | | | | | | | | |
| ^a^ α-SMA_(Invasive front of tumor)_, low, ≤4 scores; high, >4 scores | | | | | | | | | | | |
| ^b^ CD68_(Stroma)_, low, ≤600 cells; high, >600 cells | | | | | | | | | | | |
| ^c^ CD163_(Stroma)_, low, ≤600 cells; high, >600 cells | | | | | | | | | | | |

| **Supplementary Table S6. Univariate and multivariate analyses of factors associated with overall survival (OS) and disease-free survival (DFS) in the generation and validation datasets of patients with ESCC** | | | | | | | | | | | |
| --- | --- | --- | --- | --- | --- | --- | --- | --- | --- | --- | --- |
| Variables | Generation dataset | | | | |  | Validation dataset | | | | |
|  | OS | |  | DFS | |  | OS |  |  | DFS |  |
|  | HR(95%CI) | *P* |  | HR(95%CI) | *P* |  | HR(95%CI) | *P* |  | HR(95%CI) | *P* |
| **Univariate analysis** |  |  |  |  |  |  |  |  |  |  |  |
| Age (>58 *vs* ≤58) | 0.873(0.507 to 1.504) | 0.625 |  | 0.883(0.549 to 1.421) | 0.608 |  | 0.994(0.567 to 1.740) | 0.982 |  | 1.016(0.605 to 1.709) | 0.951 |
| Gender (Female vs Male) | 0.780(0.416 to 1.462) | 0.438 |  | 1.008(0.568 to 1.789) | 0.978 |  | 1.488(0.722 to 3.067) | 0.282 |  | 1.323(0.685 to 2.554) | 0.405 |
| Tumor Size |  | 0.301 |  |  | 0.747 |  |  | 0.246 |  |  | 0.211 |
| 3-5 cm vs ≤ 3 cm | 1.508(0.780 to 2.918) | 0.222 |  | 1.175(0.672 to 2.052) | 0.572 |  | 1.583(0.798 to 3.139) | 0.189 |  | 1.724(0.902 to 3.298) | 0.100 |
| >5 cm vs ≤ 3 cm | 1.748(0.832 to 3.670) | 0.140 |  | 1.269(0.669 to 2.408) | 0.465 |  | 1.956(0.862 to 4.436) | 0.108 |  | 1.793(0.831 to 3.871) | 0.137 |
| pTNM-stage (III+IV vs I+II) | 1.350(0.719 to 2.532) | 0.350 |  | 2.029(1.139to 3.614) | 0.016 |  | 2.894(1.354 to 6.185) | 0.006 |  | 1.371(0.760 to 2.474) | 0.294 |
| Molecular prognostic model |  | <0.001 |  |  | <0.001 |  |  | <0.001 |  |  | <0.001 |
| Medium-risk vs ≤ Low-risk | 4.560(2.003 to 10.381) | <0.001 |  | 2.753(1.511 to 5.017) | 0.001 |  | 2.371(1.106 to 5. 083) | 0.027 |  | 2.126(1.084 to 4.173) | 0.028 |
| High-risk vs ≤ Low-risk | 17.582(6.837 to 45.213) | <0.001 |  | 10.696(5.005 to 22.856) | <0.001 |  | 8.147(3.538 to 18.762) | <0.001 |  | 6.490(3.029 to 13.907) | <0.001 |
| **Multivariate analysis** |  |  |  |  |  |  |  |  |  |  |  |
| pTNM-stage (III+IV vs I+II) |  |  |  | 1.716(0.951 to 3.096) | 0.073 |  | 1.954(0.883 to 4.326) | 0.098 |  |  |  |
| Molecular prognostic model |  | <0.001 |  |  | <0.001 |  |  | <0.001 |  |  | <0.001 |
| Medium-risk vs ≤ Low-risk | 5.929(2.569 to 13.685) | <0.001 |  | 2.975(1.615 to 5.478) | <0.001 |  | 2.183(1.013 to 4.702) | 0.046 |  | 2.126(1.084 to 4.173) | 0.028 |
| High-risk vs ≤ Low-risk | 41.656(14.049 to 123.517) | <0.001 |  | 11.059(5.013 to 24.401) | <0.001 |  | 6.342(2.666 to 15.088) | <0.001 |  | 6.490(3.029 to 13.907) | <0.001 |
| NOTE: Multivariate analysis, Cox proportional hazards regression model. Variables were adopted for their prognostic significance by univariate analysis. | | | | | | | | | | | |


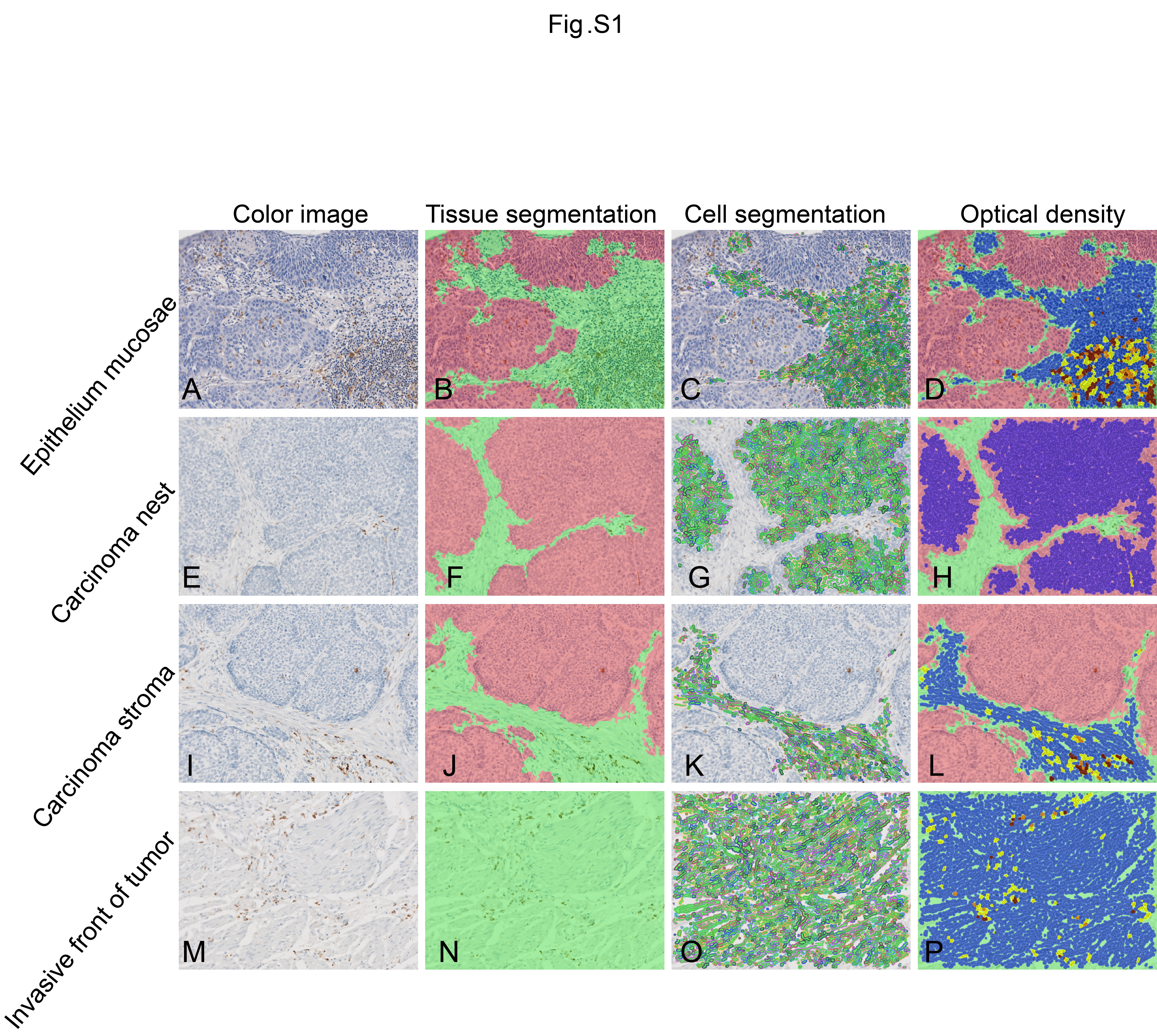


**Fig. S1.** **Representative images showing the scoring process by the automated quantitative pathology imaging system.** **(A/E/I/M)** Images of IHC staining in ESCC samples. **(B/F/J/N)** Tissue segmentation training analysis of the IHC images. **(C/G/K/O)** Cell segmentation analysis was performed to determine tumor cells. Nuclei are shown in green, and the cytoplasm for each cell is outlined in color. **(D/H/L/P)** Spectral analysis based on optical density grouped the cells into 4 categories: Blue: 0, Yellow: +, Orange: ++, and Brown: +++. The same ESCC sample is shown in A-D, E-H, I-Land M-P, respectively.


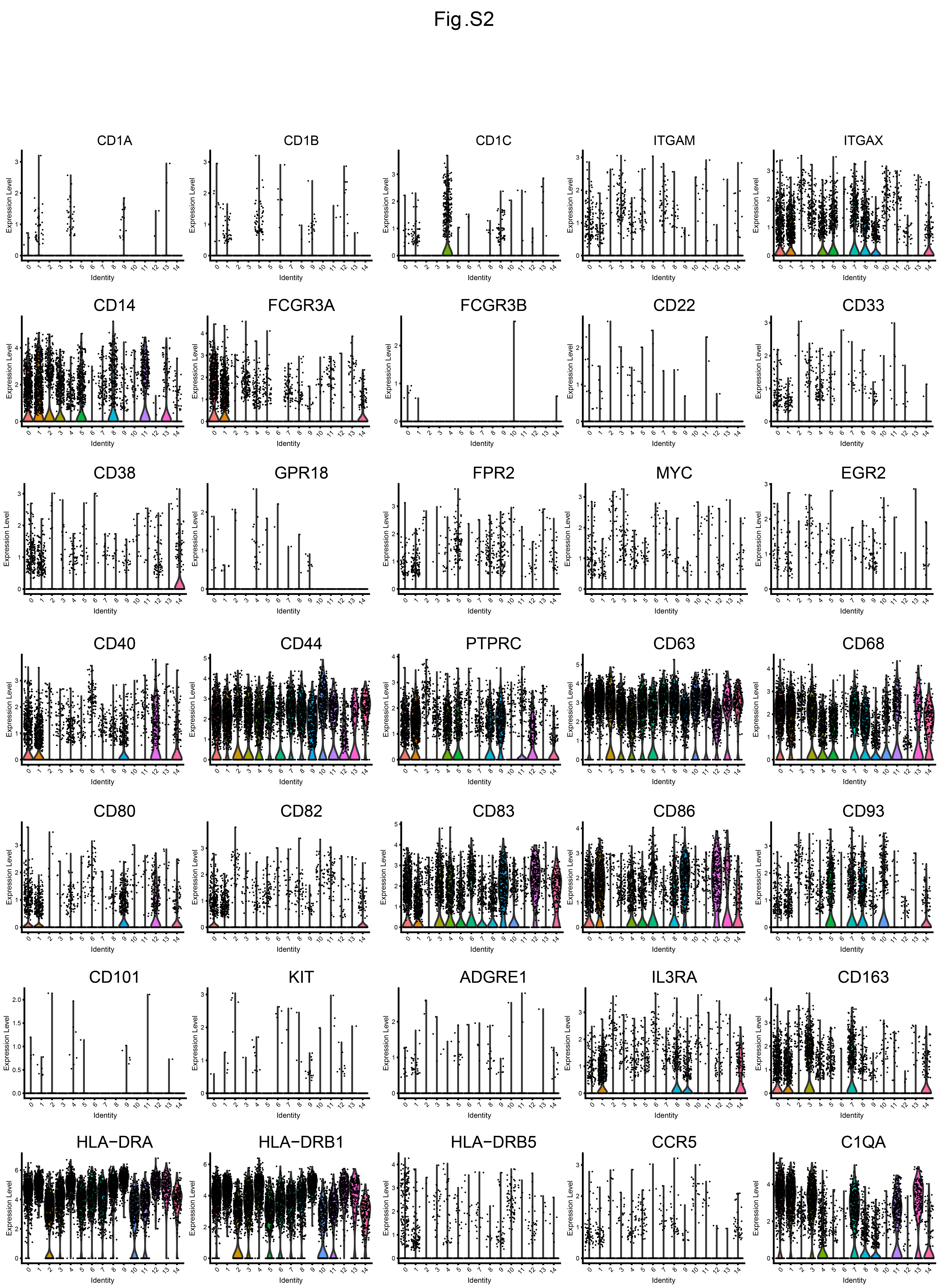


**Fig. S2**. **Violin plots displaying the expression level of representative markers in each cell cluster.**


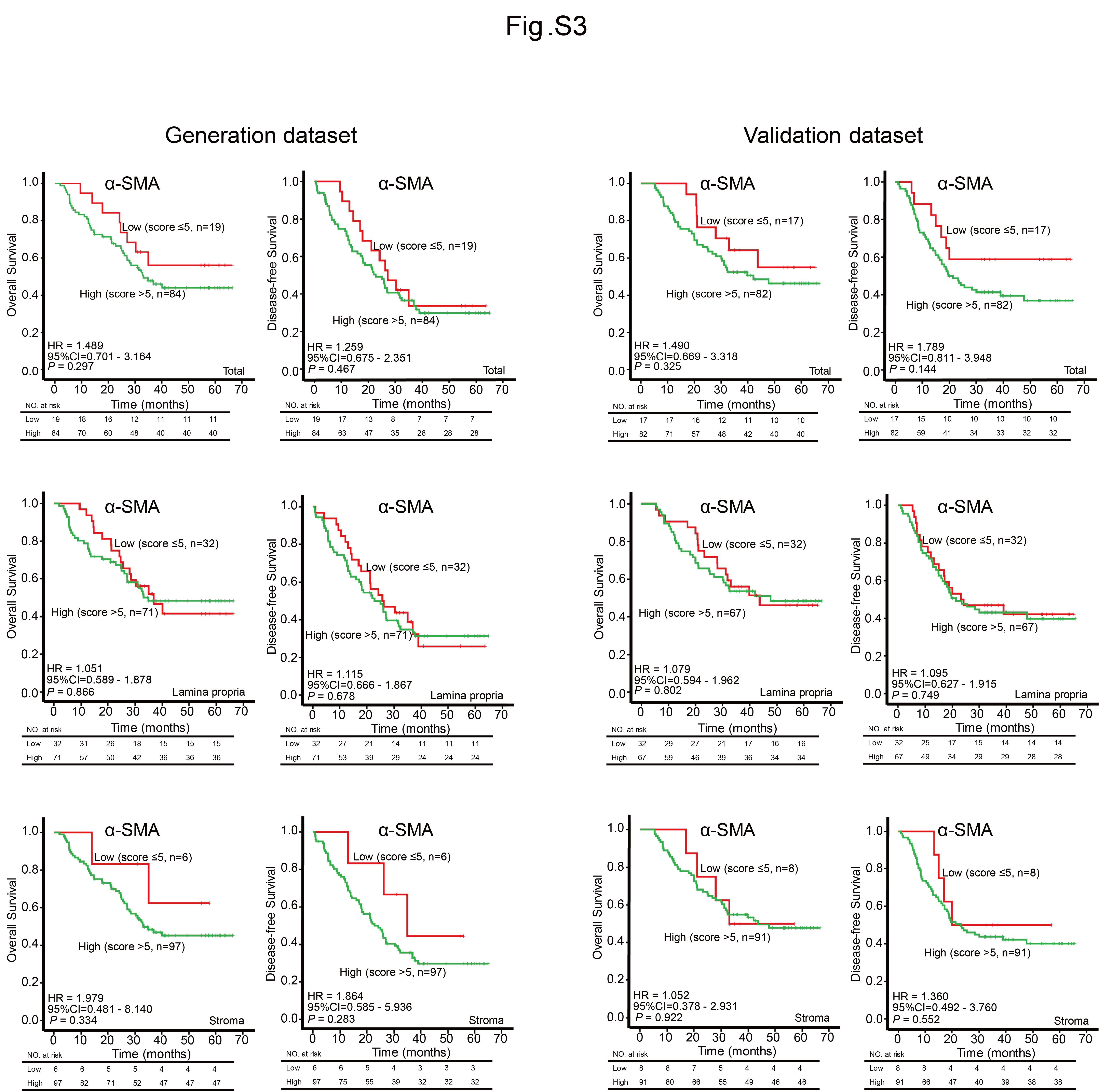


**Fig. S3**. **Kaplan-Meier survival curves for total α-SMA^+^ CAFs, lamina propria α-SMA^+^ CAFs and stromal α-SMA^+^ CAFs in the generation (n=103) and validation (n=99) dataset of patients with ESCC.**


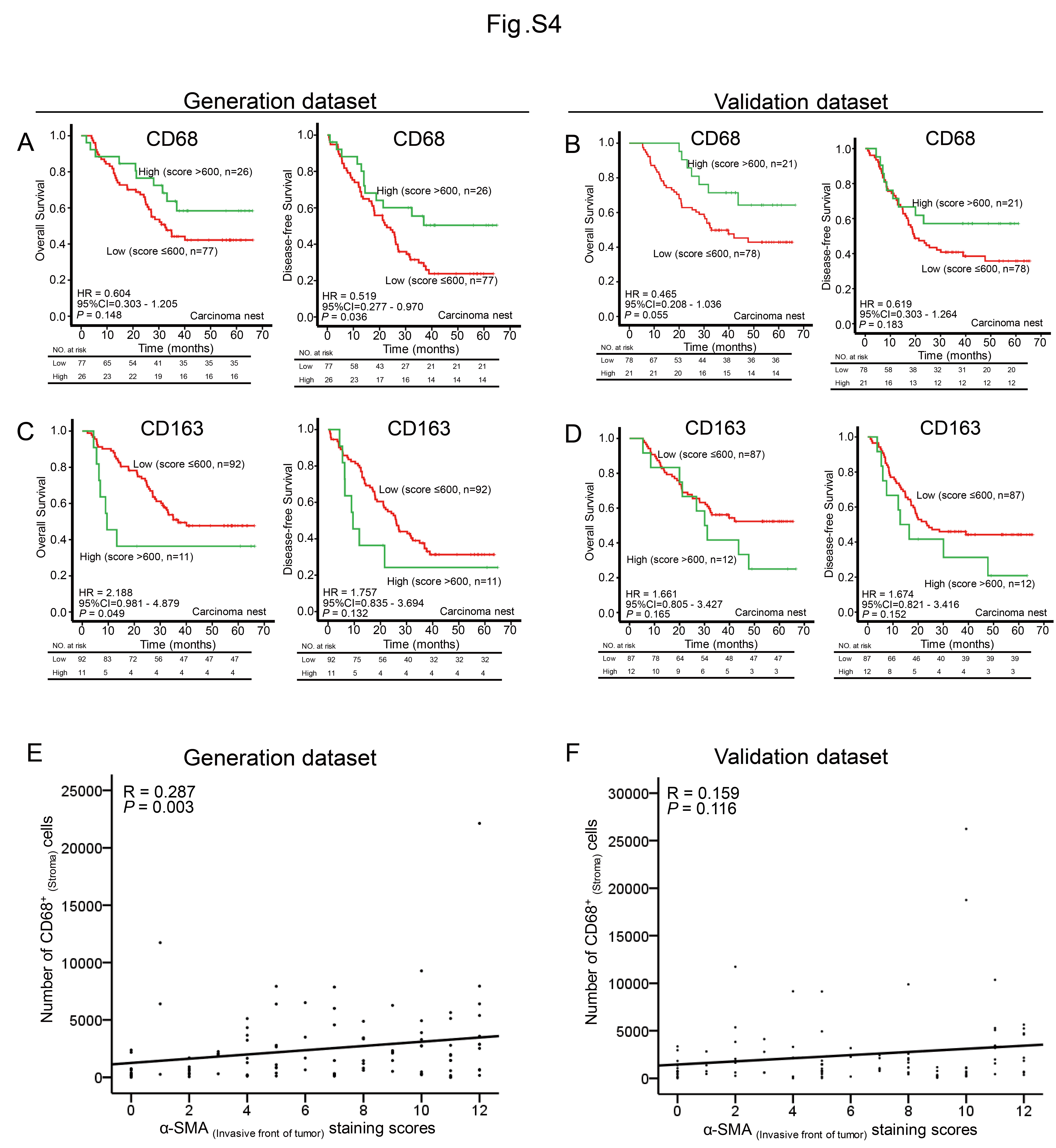


**Fig. S4. The number of intratumoral macrophages correlates with clinical outcome in ESCC patients.** **(A-D)** Prognostic values of intratumoral CD68^+^ and CD163^+^ MØs in ESCC patients in the generation and validation datasets were assessed by Kaplan-Meier survival analysis. **(E/F)** Correlation between α-SMA_(invasive front)_ and stromal CD68^+^ MØs in ESCC patients was estimated by Pearson correlation analysis.


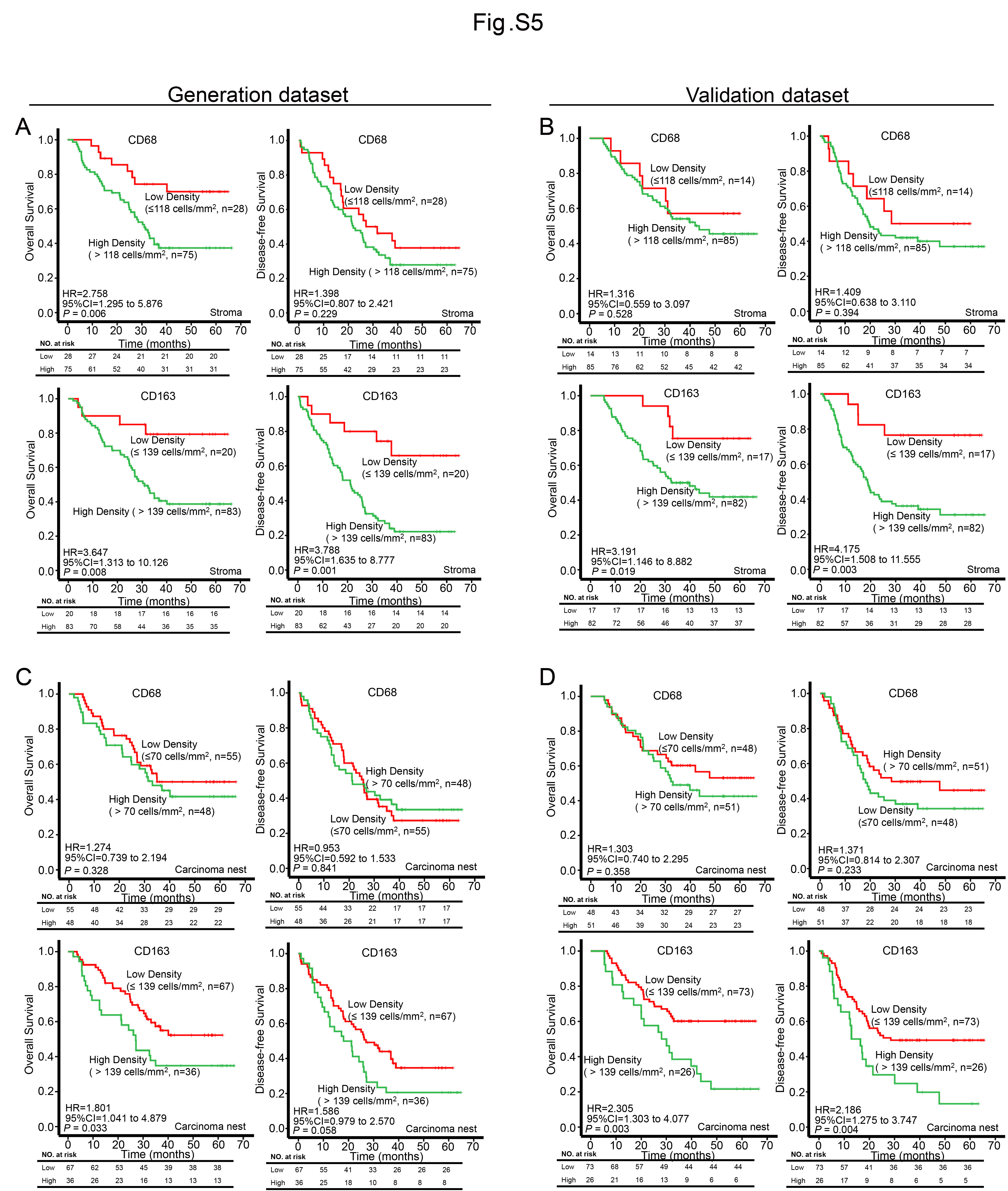


**Fig. S5. The density of CD68^+^ and CD163^+^ MØs correlates with clinical outcome in patients with ESCC. (A-D)** Prognostic values of intratumoral or stroma CD68^+^ and CD163^+^ MØs in ESCC patients of the generation and validation datasets were assessed by Kaplan-Meier survival analysis.


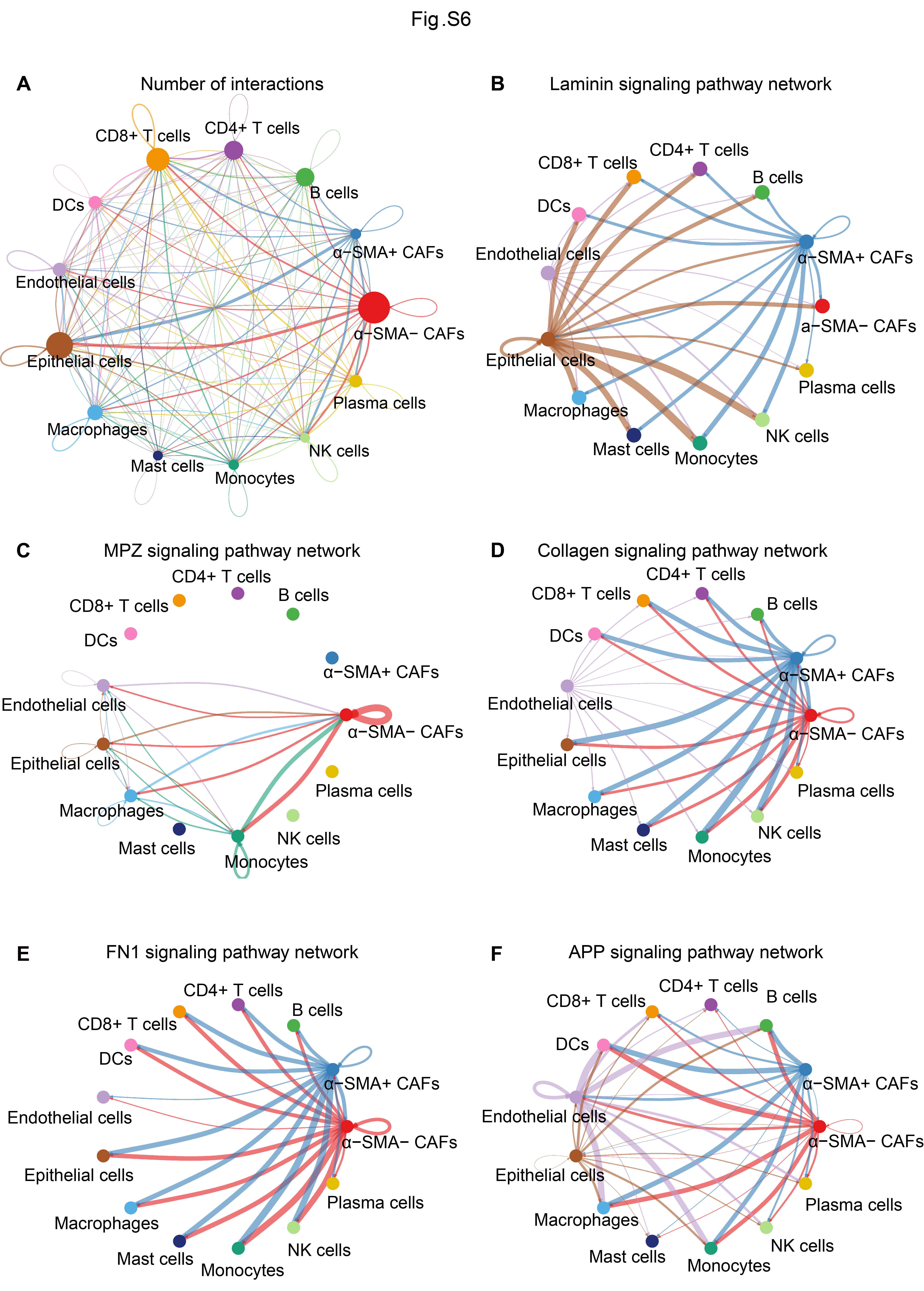


**Fig. S6**. **Crucial cell-to-cell interaction pathways among the distinct cell populations predicted by CellChat. (A)** Diagrams showing the interaction numbers in the cell cluster network. **(B-F)** Circle plots exhibiting the interactions of laminin, MPZ, collagen, FN1 and APP signaling pathway networks between CAFs and other cell clusters. Circle sizes are proportional to the number of cells in each cell type, and edge width represents the communication probability.


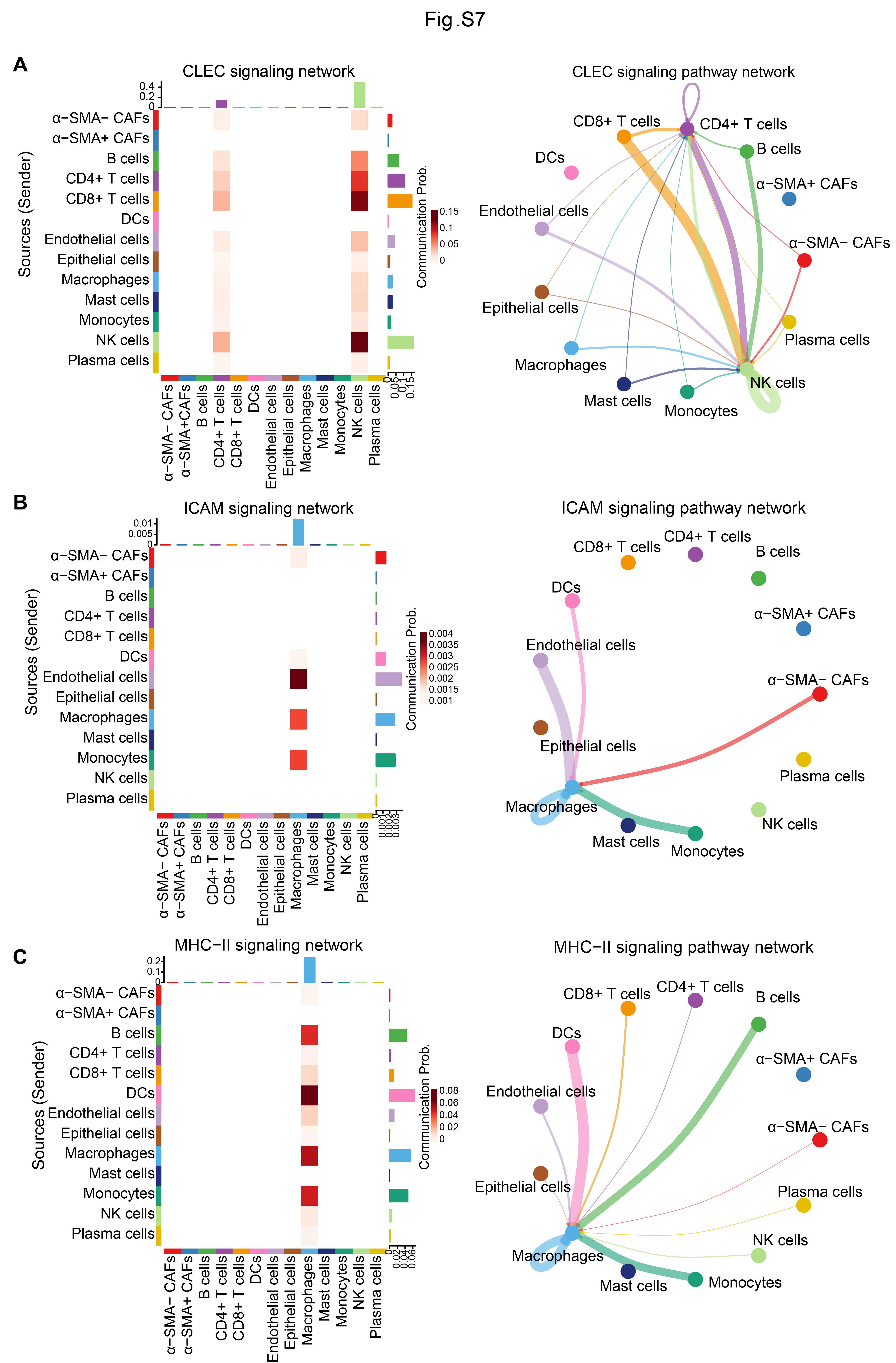


**Fig. S7**. **Cell-to-cell communications among the CAFs and other cell types. (A-C)** Heatmap and circle plots showing the intercellular communication networks. α-SMA^+^ CAFs were not found to interact with other cell clusters through the CLEC, ICAM and MHC-signaling pathways.


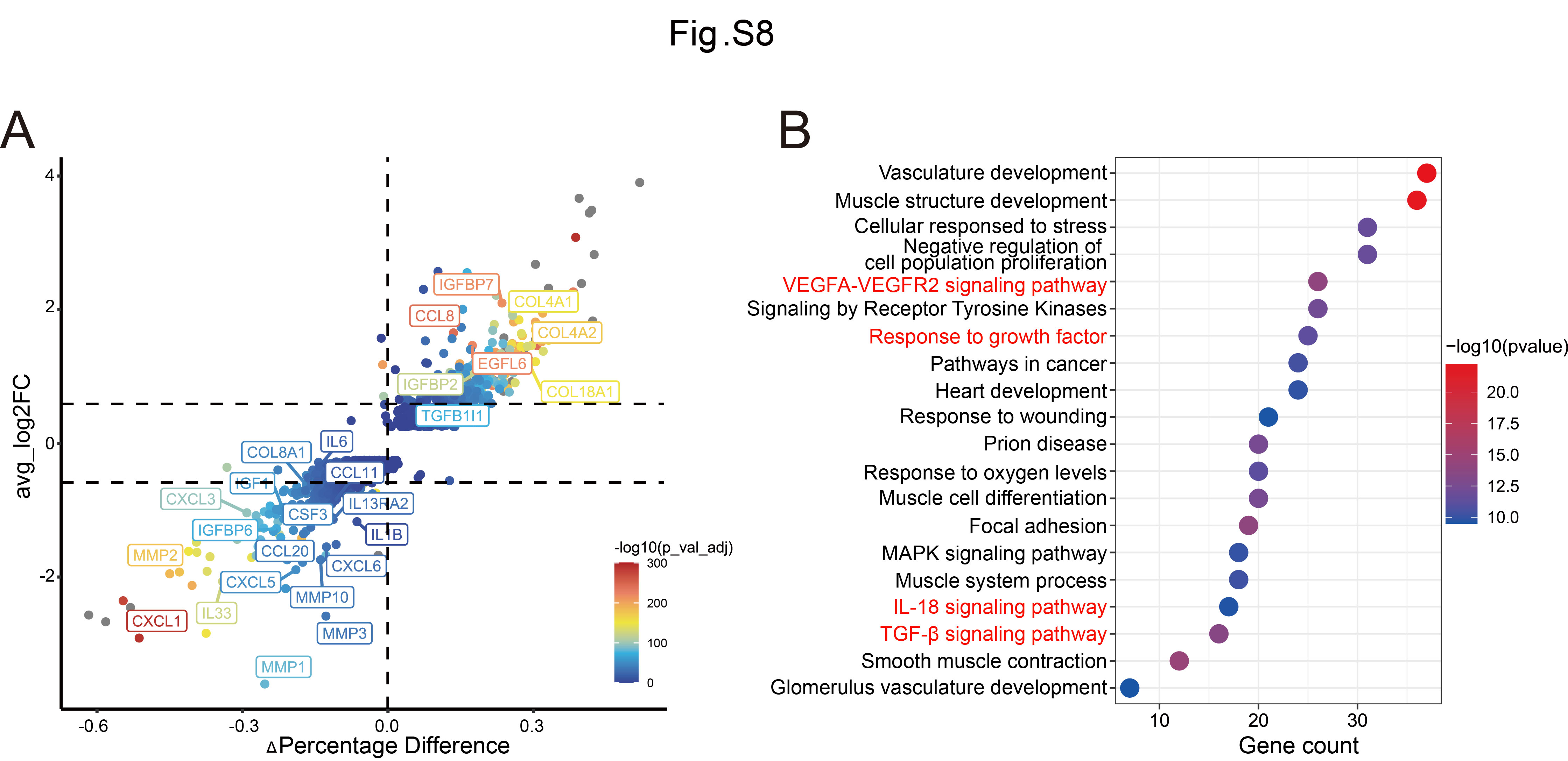


**Fig. S8**. **Differentially-expressed gene (DEG) enrichment analysis for α-SMA^+^ CAFs.** (A) Differential gene expression analysis using log-fold change in expression versus the difference in the percentage of cells expressing the gene, comparing α-SMA^+^ CAFs versus α-SMA^-^ CAFs (Δ Percentage Difference). Genes labeled have log-fold change > 1, Δ Percentage Difference > 20% and adjusted *p*-value from the Wilcoxon rank sum test <0.05. (B) Enrichment analysis of the genes that are highly expressed (fold change > 1.5, P<0.05) in α-SMA^+^ CAFs by Metascape.
